# Spiking and Membrane Properties of Rat Olfactory Bulb Dopamine Neurons

**DOI:** 10.3389/fncel.2020.00060

**Published:** 2020-03-20

**Authors:** Kirill S. Korshunov, Laura J. Blakemore, Richard Bertram, Paul Q. Trombley

**Affiliations:** ^1^Program in Neuroscience, Florida State University, Tallahassee, FL, United States; ^2^Department of Biological Science, Florida State University, Tallahassee, FL, United States; ^3^Department of Mathematics, Florida State University, Tallahassee, FL, United States

**Keywords:** dopamine, olfactory bulb, electrophysiology, membrane properties, H-current, Na^+^ current, ramp protocols

## Abstract

The mammalian olfactory bulb (OB) has a vast population of dopamine (DA) neurons, whose function is to increase odor discrimination through mostly inhibitory synaptic mechanisms. However, it is not well understood whether there is more than one neuronal type of OB DA neuron, how these neurons respond to different stimuli, and the ionic mechanisms behind those responses. In this study, we used a transgenic rat line (hTH-GFP) to identify fluorescent OB DA neurons for recording via whole-cell electrophysiology. These neurons were grouped based on their localization in the glomerular layer (“Top” vs. “Bottom”) with these largest and smallest neurons grouped by neuronal area (“Large” vs. “Small,” in μm^2^). We found that some membrane properties could be distinguished based on a neuron’s area, but not by its glomerular localization. All OB DA neurons produced a single action potential when receiving a sufficiently depolarizing stimulus, while some could also spike multiple times when receiving weaker stimuli, an activity that was more likely in Large than Small neurons. This single spiking activity is likely driven by the Na^+^ current, which showed a sensitivity to inactivation by depolarization and a relatively long time constant for the removal of inactivation. These recordings showed that Small neurons were more sensitive to inactivation of Na^+^ current at membrane potentials of −70 and −60 mV than Large neurons. The hyperpolarization-activated H-current (identified by voltage sags) was more pronounced in Small than Large DA neurons across hyperpolarized membrane potentials. Lastly, to mimic a more physiological stimulus, these neurons received ramp stimuli of various durations and current amplitudes. When stimulated with weaker/shallow ramps, the neurons needed less current to begin and end firing and they produced more action potentials at a slower frequency. These spiking properties were further analyzed between the four groups of neurons, and these analyses support the difference in spiking induced with current step stimuli. Thus, there may be more than one type of OB DA neuron, and these neurons’ activities may support a possible role of being high-pass filters in the OB by allowing the transmission of stronger odor signals while inhibiting weaker ones.

## Introduction

Olfaction is central to the perception of chemical environments and is a necessary sensory system for the survival of most animals. The OB is the first region of the brain to receive and modify odor signals before sending them to higher brain regions. In many ways similar to the retina, the OB accomplishes its tasks by utilizing different subtypes of neurons embedded in distinct laminae. The neuronal circuitry and synaptic activities within the OB are complex. Chemical odors are first transduced by the OSNs in the olfactory epithelium. OSNs form glutamatergic axodendritic synapses (Berkowicz et al., 1994; Ennis et al., 1996) with interneurons of the GL and the main output neurons of the OB, mitral and tufted cells (M/TCs) (Pinching and Powell, 1971; Bardoni et al., 1996a, b; Kosaka et al., 1997; Keller et al., 1998). The interneurons found in the GL are collectively termed JGCs, which can be divided into three types: PGCs, SACs, and ETCs (Golgi, 1875; Pinching and Powell, 1971; Shepherd, 1972; Shepherd et al., 2011; Nagayama et al., 2014). As the odor signal is being transmitted to the M/TCs, the JGCs modify the signal by the release of neurotransmitters such as glutamate, GABA, and DA.

Endogenous to the GL, DA-releasing JGCs are localized around the spherical, dense neuropil structures called glomeruli. DA is expressed in 10–16% of all JGCs; this corresponds to roughly 88,000 neurons in the GL of the OB of the mouse and roughly 100,000 neurons in the GL of the rat (McLean and Shipley, 1988; Panzanelli et al., 2007; Parrish-Aungst et al., 2007). The DA neuron population in the OB is estimated to be the largest in the entire brain (Cave and Baker, 2009). These neurons mainly make inhibitory contacts with the OSNs and the apical dendrites of M/TCs (Nickell et al., 1994; Hsia et al., 1999; Berkowicz and Trombley, 2000; Ennis et al., 2001; Davila et al., 2003; Vaaga et al., 2017). Functionally, these DA neurons are important for increasing odor resolution by simultaneously increasing odor discrimination and decreasing odor noise (Wilson and Sullivan, 1995; Ennis et al., 2001; Tillerson et al., 2006). Thus, the gating mechanisms of OB DA neurons are crucial, but it is not fully understood how these neurons respond to specific signal stimuli.

Recent studies show that OB DA neurons fall into two categories: larger neurons possessing an axon and smaller neurons that are anaxonic (Chand et al., 2015; Galliano et al., 2018). These results support earlier reports (Halász et al., 1981; Pignatelli et al., 2005; Kosaka and Kosaka, 2007, 2008, 2009) and reviews (Kosaka and Kosaka, 2011, 2016; Pignatelli and Belluzzi, 2017) describing two distinct sizes of OB DA neurons. What is/are the potential identities and locations of these small and large DA neurons? Based on different neuronal features, many studies often categorize OB DA neurons as being either PGCs (Kosaka et al., 1995, 1997, 1998; Kosaka and Kosaka, 2007; Parrish-Aungst et al., 2007) or SACs (Kiyokage et al., 2010; Liu et al., 2013; Cockerham et al., 2016; Bywalez et al., 2017), with SACs having a slightly larger soma size than PGCs (Pinching and Powell, 1971; Nagayama et al., 2014). A subgroup of potential DAergic PGCs were identified as the “Type-1” PGCs, which express TH (the rate-limiting enzyme present in all DA neurons) and receive excitatory input from the “ON Zone,” corresponding to the area between the middle and the superficial (ONL/GL) border of the glomerulus (Kosaka et al., 1995, 1997, 1998; Kosaka and Kosaka, 2005, 2007). A potential DAergic population of SACs provide the most common source of interglomerular projections in the OB (Aungst et al., 2003; Kiyokage et al., 2010), thus, may correspond to the axonic DA neurons, which are mostly found in the deeper (closer to the EPL) portion of the GL (Galliano et al., 2018). Therefore, to distinguish between these two potential types of OB DA neurons, we used whole-cell electrophysiology to investigate differences in the membrane properties of OB DA neurons based on their laminar localization in the GL and size (neuronal area).

The response of a neuron to artificial stimuli can be indicative of both how that neuron responds to natural stimuli and the functional outcomes in the neuronal circuit. Surprisingly, there is a lack of information regarding how OB DA neurons respond to artificial stimuli. Therefore, another focus of this study was to determine the firing and gating properties of OB DA neurons in response to evoked current step stimuli. Further, the ionic currents that directly and indirectly contribute to spiking properties – I_Na_ and the non-specific cation I_H_, respectively (Pignatelli et al., 2013; Iseppe et al., 2016) – were examined in these neurons. To potentially further distinguish between types of OB DA neurons, these properties were also studied in neurons categorized according to laminar (GL) localization and neuronal area.

Lastly, the signal processing properties of OB DA neurons were investigated. These neurons are mostly inhibitory and, upon activation, release DA and the inhibitory neurotransmitter GABA, which most OB DA neurons co-express (Kosaka et al., 1985, 1995; Gall et al., 1987; Baker et al., 1988; Maher and Westbrook, 2008; Borisovska et al., 2013; Liu et al., 2013, 2016). These inhibitory actions could increase odor discrimination through the activity of the D_2_ receptor (Tillerson et al., 2006). It has been shown that higher odor concentrations increase odor discrimination (Wei et al., 2006). To investigate whether the OB DA neurons contribute to this, we used a whole-cell current-clamp recording protocol that injected ramps of current into the neurons, with variable ramp slopes. Unlike the conventional step protocols, these ramp protocols are more akin to the summation properties of natural stimuli. In combination with the conventional step stimulations, the ramp stimuli allowed us to determine how responsive OB DA neurons are to strong and weak stimuli. These responses were also differentiated between OB DA neurons based on their GL localization and neuronal area.

As many previous studies have characterized OB DA neurons in transgenic mice (Pignatelli et al., 2005, 2009, 2013; Puopolo et al., 2005), the last goal of this study was to determine these properties in the rat. Rats offer some clear advantages over mice (e.g., easier to handle, lower susceptibility to stress, larger brain size facilitates brain surgery and imaging) and are better models for the study of some human behaviors and conditions (Ellenbroek and Youn, 2016). For these experiments, we used a transgenic rat line – the TH green fluorescent protein (hTH-GFP) line (Iacovitti et al., 2014) – which expresses GFP in all TH + (DA) neurons in the OB and other regions of the brain. The advent of this transgenic rat model has important implications to future research and facilitates the exploration of species differences (see section “Discussion”). Whereas previous research in the rat OB characterized the biophysical properties of JGCs without determining the cell type (DA or other) (e.g., Puopolo and Belluzzi, 1998), our transgenic rat model allowed us to directly examine the electrophysiological properties of fluorescent OB DA neurons and to determine potential differences between rat and mouse OB DA neurons.

Overall, our results show that OB DA neurons may have spiking properties that differ from those of other OB neurons. These spiking properties, along with their membrane properties, I_H_, and gating properties, may differ between neurons based on the neuronal area, but not necessarily the localization of these neurons in the GL. Given that OB DA neurons appear to be more responsive to weaker stimuli and are inhibitory, these results also imply that these neurons act as high-pass filters in the OB. Additionally, these spiking properties are characteristic of DA neurons of rats, but not mice. These findings provide further insight not only to the identity of the OB DA neurons, but also to their signal processing properties that allow them to respond to different odor signals to properly process information in the rat OB.

## Materials and Methods

### Animals

Transgenic hTH-GFP Sprague Dawley rats (Iacovitti et al., 2014) were used for all experiments (Taconic Biosciences, United States). Rats were housed in an animal vivarium facility at Florida State University, exposed to a 12-h light and dark cycle, and provided *ad libitum* access to food and water. All experiments were carried out in accordance with the current edition (8th) of the National Institutes of Health Guide for the Care and Use of Laboratory Animals, and the Florida State University Institutional Animal Care and Use Committee approved all procedures.

### Olfactory Bulb Dissection

Rat OB tissue slices were prepared as previously described (Blakemore et al., 2006). Rats between the ages of P12 and P22 were used. A total of 83 rats were used for these experiments (approximately 2 rats for each day of recording). Animals were anesthetized with isoflurane (Henry Schein Animal Health, Dublin, OH, United States) and were decapitated. OBs were harvested in ice cold, oxygenated (95% O_2_, 5% CO_2_) aCSF with sucrose (sucrose aCSF). The makeup of the sucrose aCSF solution is as follows (in mM): 83 NaCl, 2.5 KCl, 26.2 NaHCO_3_, 1 NaH_2_PO_4_, 0.5 CaCl_2_, 3.3 MgCl_2_, 22 glucose, and 72 sucrose. OBs were glued onto a metal pedestal using cyanoacrylate and 300-μm thick horizontal slices were cut with a Vibratome (St Louis, MO, United States) in ice-cold sucrose aCSF solution. Slices were gently transferred to a holding chamber, incubated in 35°C-oxygenated aCSF solution for 30 min, and then stored at 20–24°C until use. The makeup of the aCSF solution is as follows (in mM): 125 NaCl, 2.5 KCl, 25 NaHCO_3_, 1.25 NaH_2_PO_4_, 2 CaCl_2_, 1 MgCl_2_, and 25 glucose. Tissue slices were then transferred to a recording chamber for all electrophysiology experiments.

### Electrophysiology

For all electrophysiology experiments, 300-μm horizontal OB slices were used for recordings in whole-cell current- and voltage-clamp modes. A Multiclamp 700B amplifier (Molecular Devices, Axon Instruments, San Jose, CA, United States), ITC-18 digitizer (Instrutech, Longmont, CO, United States), and AxographX acquisition software (John Clements) were used for all data acquisition. Neurons were visualized with a Leica DMLFS fluorescent microscope (Meyer Instruments, Houston, TX, United States) and a Hitachi HV-D30 camera (B&H, NY, United States).

Borosilicate glass (World Precision Instruments, Sarasota, FL, United States) was pulled into electrodes with a final resistance of 4–6 MΩ. The intracellular solution for most whole-cell recordings was composed of the following (in mM): 125 KMeSO_4_, 0.025 CaCl_2_, 2 MgCl_2_, 1 EGTA, 2 Na_2_ATP, 0.5 NaGTP, and 10 HEPES. Neurons were constantly perfused with an oxygenated aCSF solution at a rate of 1 ml per minute. In total, we recorded from 140 neurons from OB slices. Extracellular drugs were delivered by bath perfusion. We used 25 μM CdCl_2_ to inhibit voltage-gated calcium channels (Ca_v_) for I_Na_ analyses. In addition, 5 μg/ml of propidium iodide was used to visualize the OB layers, which was incubated with post-recorded slices for 2 h in 20–24°C before being imaged with a Leica DMLB fluorescent microscope (Meyer Instruments, Houston, TX, United States) and an Andor camera (Andor, Oxford Instruments, Europe), with the NIS Elements AR 3.2 software (Nikon, Melville, NY, United States).

### Calculating Membrane Properties

Membrane properties of OB DA neurons were compared based on their localization in the GL (“Top” vs. “Bottom”) and neuronal areas (“Large” vs. “Small” neurons). The localization of DA neurons in the GL was distinguished visually before targeting them for whole-cell electrophysiology recordings. “Top” DA neurons were identified as the fluorescent neurons in the “upper half” (the area between the center and the ONL/GL border) of their respective glomeruli. “Bottom” DA neurons were identified as the fluorescent neurons in the “bottom half” (the area between the center and the GL/EPL border) of their respective glomeruli. We recorded from a total of 94 neurons (45 Top and 49 Bottom) categorized in this manner. (The rest of the recorded neurons were not identified by their localization or neuronal area and were thus excluded from these and subsequent calculations).

Of the total of 140 neurons from which we recorded in slice, we determined neuronal areas (“areas”) for 87 of these neurons. For the purpose of analysis, these neurons were categorized according to size and separated into thirds. As large versions of the “Small” neurons could overlap with small versions of the “Large” neurons, we eliminated the middle group of neurons from this analysis to reduce misidentification of cells. Therefore, one-third (*n* = 29) of the neurons with the largest areas (3390 to 4890 μm^2^) and one-third (*n* = 27) of the neurons with the smallest areas (1589 to 2610 μm^2^) were used for calculations and comparisons of membrane properties based on neuronal area. All membrane properties were calculated from current-clamp voltage traces.

Membrane resistance was calculated by analyzing the hyperpolarizing voltage deflection in response to current injection (−10, −25, −50, or −75 pA step) using V = IR. The time constant (τ) was calculated by analyzing the amount of time it took for the neuron to hyperpolarize to 63% of its total voltage step. Capacitance was then derived using τ = RC. From the capacitance, the neuronal area was calculated by C = AC_*m*_. The C_*m*_ (specific capacitance) was previously determined to be 1.0 $\frac{\mu \,F}{c\,m\,2}$ (Hodgkin and Huxley, 1952; Holohean et al., 1996; Gentet et al., 2000), which was converted to 0.01 $\frac{p\,F}{\mu \,m\,2}$the value used for these calculations. The neurons’ action potential thresholds were determined through specific protocols. The first protocol injected 40, 3-ms depolarizing currents, at 1.5 s increments, with each incremental injection being 10 pA more depolarizing than the last (beginning with 10 pA and ending with 400 pA). The action potential threshold was defined as the amount of current that produced a voltage spike distinct from an Ohmic response. If the first protocol did not produce spiking in a neuron, a second protocol was used with the same specifications, except the injections were increased to increments of 20 pA (beginning with 20 pA and ending with 800 pA). Finally, voltage sag ratios were calculated by analyzing the minimum (V_i_) and final (V_f_) membrane voltages of a hyperpolarization step. The V_i_ is the value of the voltage drop before the depolarization sag, while the V_f_ is the voltage value at the very end of a hyperpolarizing stimulus (voltage sag ratio = $\frac{V_{i}-V_{f}}{V_{i}}$). All calculations were made on voltage drops produced by −25, −50, and −75 pA stimuli.

### Na^+^ Current Properties

For the recording of the I_Na_, the intracellular recording solution consisted of the following (in mM): 125 CsCl, 2 MgCl_2_, 1.1 EGTA, 2 ATP, 0.5 GTP, and 10 HEPES. To isolate these inward currents from the inward I_Ca_^2+^, recordings were made in the presence of 25 μM CdCl_2_ in the extracellular aCSF solution. All I_Na_s were elicited by depolarizing the neuronal membrane with 200-ms, 10-mV voltage steps. I_Na_ was identified as a transient fast-activating and inactivating inward current that would last no longer than 10 ms. The current-voltage I_Na_ curve was constructed by taking the peak current values at each depolarization step (ranging from −80 to 30 mV). To calculate the inactivation (h∞) curve, the neuronal membrane received 7 50-ms pre-pulse voltage steps (ranging from −90 to −30 mV, in 10 mV increments), followed by a 100-ms 80 mV depolarizing step. The currents that resulted at the 80-mV step were used to calculate the inactivation curve. The I_Na_ with the largest amplitude, occurring when the membrane went from −90 to 80 mV, was used as a reference peak. At this point, the peak of each subsequent current (−80, −70 mV, etc.) was divided by the maximum peak, and the resulting ratios showed how much voltage-gated Na^+^ (Na_v_) channels were inactivated at certain membrane potentials. The half-inactivation is presented in this paper as the membrane potential (I_Na_ Peak/I_Na_ Peak (Max)) = 0.5. This inactivation curve shows the cumulative peak from 23 neurons. Each neuron also had an individual inactivation curve constructed, where their individual membrane potentials at half inactivation values were derived and compared. Two additional inactivation plots were constructed for Top vs. Bottom and Large vs. Small DA neurons, which were taken from the pooled sample of 23 neurons. To derive the amount of time it would take to remove inactivation from 63% of Na_v_s, we constructed an IPI curve. This curve was derived by depolarizing each neuron with two 20-ms 60 mV voltage steps, with each pair of pulses separated by increasing intervals (0.5, 1, 3, 5, 7.5, 10, 12.5, 15, and 50 ms). At each IPI, the peak of current 2 (from the second 60 mV pulse) was divided by the peak of current 1 (from the first 60 mV pulse). The resulting ratio shows how the increasing durations of IPIs remove the inactivation of Na_v_. The amount of time it would take to remove inactivation from 63% of these channels was derived when the curve crossed I_Na_ Peak 2/I_Na_ Peak 1 = 0.63. The cumulative curve was constructed from 25 neurons. Subsequently, each neuron also had its own IPI curve constructed, and their individual times to remove inactivation from 63% of Na_v_s were derived and compared. Two additional removal of inactivation plots were constructed for Top vs. Bottom and Large vs. Small DA neurons, which were taken from the pooled sample of 25 neurons.

### Ramp Protocols

For all analyses involving ramp stimuli, the “ON current” and “OFF current” were found at the beginning and ending of action potential firing, respectively. The interspike period (Δt) was calculated as the time between two consecutive action potentials, with Δt_1_ = the time between the first and second action potential, Δt_2_ = the time between the second and third action potential, and so on. Individual spike frequencies (*f*) were determined by taking the inverse of each Δt.

### Data Analysis

For all statistical and graphical analyses, GraphPad Prism (version 8.2.1; La Jolla, CA, United States) was used. All data are presented as mean ± SEM. Homogeneity of variance was determined via the *F*-test. The normality of residuals was checked with the Kolmogorov-Smirnov and Shapiro-Wilk tests. Residuals were considered normally distributed if *p*-Values were >0.05. Additionally, the residual and Q-Q plots were visualized to confirm residuals’ normality. As the sampled distributions had normal distributions and equal variances, unpaired *t*-tests were used to determine whether mean values for membrane properties differed based on two groups of independent categorical variables – GL localization (“Top” vs. “Bottom” of GL) or neuronal area (“Large” vs. “Small” neurons). These results are presented as *t*(df) = x.xx, *p* = 0.xx. Statistically significant values are represented as any *p*-Value less than 0.05. ^∗^ = *p* < 0.05; ^∗∗^ = *p* < 0.01; ^∗∗∗^ = *p* < 0.001; and ^****^ = *p* < 0.0001.

For the ramp experiments, we transformed all the results into log-log plots by taking the logarithms of the *x*-Values (ramp slopes) and the *y*-Values (ON/OFF currents, spiking frequencies, and spike numbers). This transformation linearized the data, indicating that they are distributed as power functions, *y* = 10*^*b*^*⋅x*^*m*^*, where *b* is the y-intercept and *m* is the slope of the transformed data. With the data now linearized, we compared the slopes of each group (Top vs. Bottom and Large vs. Small DA neurons) using a simple linear regression analysis, after confirming that the criteria for normality (as described above) were met. A significant difference (*p* < 0.05) in *m* between different DA groups indicates a significantly different responses to changes in ramp slopes.

## Results

### Visualization and Glomerular Localization of Rat Olfactory Bulb Dopamine Neurons

**FIGURE 1:**
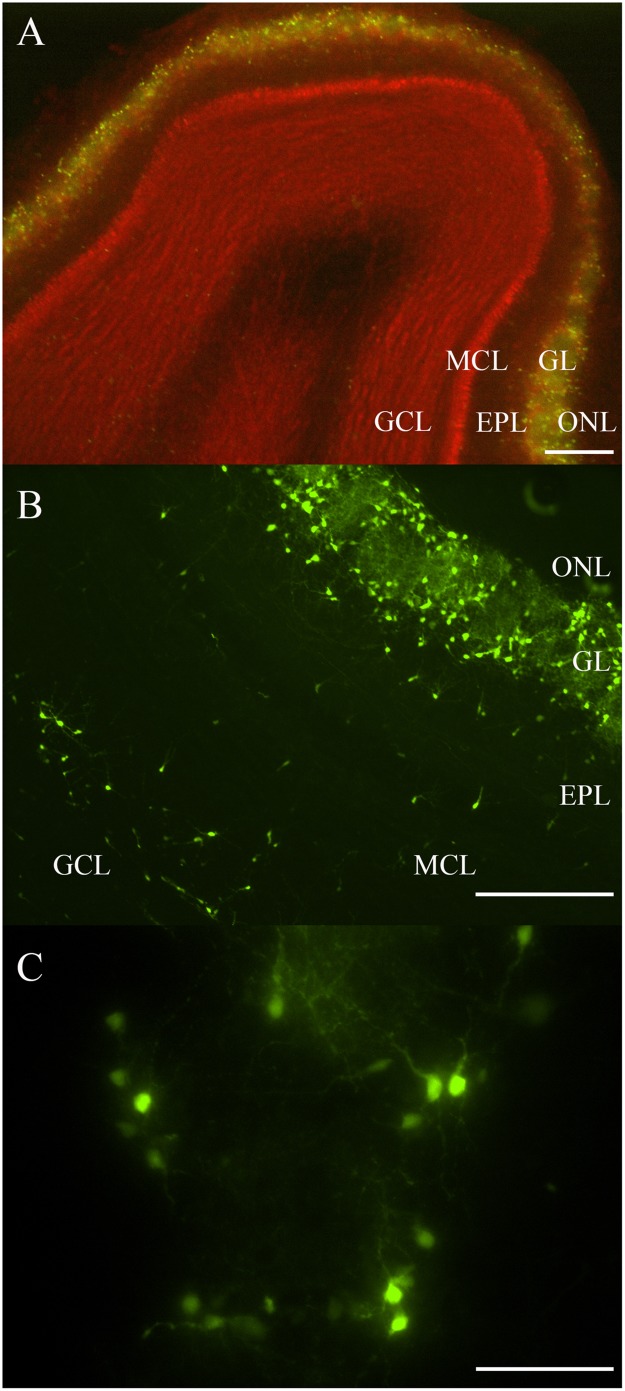
Rat OB and its endogenous DA neurons. **(A)** A horizontal OB slice with green fluorescent TH-GFP neurons localized to the GL. Discreet layers – EPL, MCL, and GCL – deep to the GL were also emphasized with propidium iodide (red). Scale bar represents 200 μm. **(B)** A higher magnification of another OB slice with fluorescent DA neurons localized mostly to the GL, but also some neurons in the EPL, MCL, and GCL. Scale bar represents 100 μm. **(C)** A single spherical glomerulus with fluorescent DA neurons around its circumference. Scale bar represents 50 μm.

Most fluorescent OB DA neurons were localized to the GL (Figures 1A–C). Some DA neurons were also expressed in layers deep to the GL, including the EPL, MCL, and GCL (Figure 1B). Neurons expressed in layers deep to the GL are likely the neonatal and adult-born DA neurons that are migrating from the subventricular zone and rostral migratory stream to their final destination within the GL (Betarbet et al., 1996; Baker et al., 2001; Pignatelli et al., 2009). Roughly 1,700 TH-positive neurons were previously reported in the EPL (Parrish-Aungst et al., 2007). These OB DA neurons showed a variety of morphologies, including multipolar and bipolar shapes, with varying soma sizes (Figures 1B,C). Some areas in the GL have DA neurons that are equally distributed around their respective glomeruli’s circumferences (Figures 1B,C). We determined whether these neurons express differences that may account for them being more than one OB DA neuron subtype.

### Membrane Properties

**FIGURE 2:**
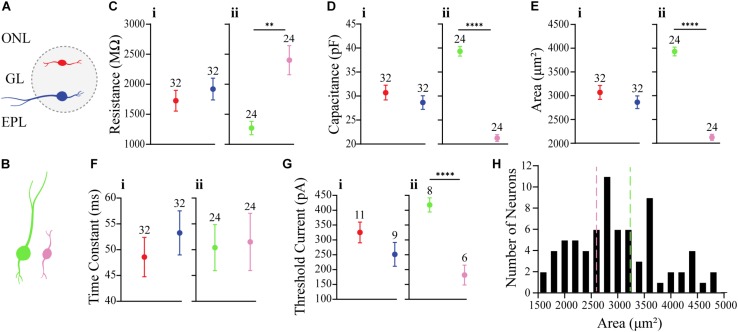
Comparison of membrane properties between OB DA neurons based on their localization in the GL and neuronal area. **(A)** Top, red neurons = closer to ONL; Bottom, blue neurons = closer to the EPL. **(B)** Large, green neurons = 3390 to 4890 μm^2^; Small, pink neurons = 1589 to 2610 μm^2^. All data represented as mean ± SEM. **(C)** For membrane resistances (in MΩ), there was no significant difference (*n* = 64, *p* = 0.4181, **Ci**) between neurons based on their glomerular localization, but there was a significant difference (*n* = 48, *p* = 0.0001**, **Cii**) based on neuronal area. **(D)** For membrane capacitance (in pF), there was no significant difference (*n* = 64, *p* = 0.2979, **Di**) between neurons based on their glomerular localization, but there was a significant difference (*n* = 48, *p* < 0.0001****, **Dii**) based on neuronal area. **(E)** For neuronal areas (in μm^2^), like capacitance, there was no significant difference (*n* = 64, *p* = 0.2979, **Ei**) based on glomerular localization. **(F)** For time constants (in ms), there was no significant difference (*n* = 64, *p* = 0.4153, **Fi**) between neurons based on their glomerular localization, and no significant difference (*n* = 48, *p* = 0.8786, **Fii**) between neurons based on neuronal area. **(G)** For action potential thresholds (in pA), there was no significant difference (*n* = 20, *p* = 0.1792, **Gi**) between neurons based on their glomerular localization, but there was a significant difference (*n* = 14, *p* < 0.0001****, **Gii**) between neurons based on area. **(H)** Frequency distribution of neuronal areas of DA neurons (2996 ± 94.72 μm^2^, *n* = 73). Pink and green dashed borders are used to distinguish Small and Large neurons, respectively.

It is unclear whether the properties of OB DA neurons expressed in the superficial half of the glomerulus (closer to the ONL; “Top,” red neurons) differ from those expressed in the deeper half of the glomerulus (closer to the EPL; “Bottom,” blue neurons) (Figure 2A). Such differences may be indicative of different neuronal subtypes, such as PGCs and SACs. We compared membrane resistance, capacitance, neuronal areas, time constants, and action potential thresholds between DA neurons localized to the upper/”top” and lower/”bottom” portions of their respective glomeruli. In the following sections, we also compared the properties of ionic currents (I_Na_, I_H_, and ON and OFF currents) and spiking properties (spiking frequencies and number of spikes) between these groups of DA neurons. For this section, we compared the membrane properties of 64 recorded neurons (32 “Top,” 32 “Bottom,” and 9 neurons not identified by their localization, which were not included in the “Top” vs. “Bottom” analyses) (Table 1).

**TABLE 1:** Membrane properties of recorded OB DA neurons based on glomerular localization.

	Top of glomerulus	Bottom of glomerulus	All neurons
Membrane resistance (MΩ)	1725 ± 173.5	1926 ± 174.5	1742 ± 113.5
Membrane capacitance (pF)	30.71 ± 1.44	28.64 ± 1.33	29.96 ± 0.95
Neuronal area (μm^2^)	3071 ± 144.1	2864 ± 133.4	2996 ± 94.72
Time constant (ms)	48.57 ± 3.82	53.26 ± 4.26	49.27 ± 2.7
Action potential threshold (pA)	325.2 ± 34.80	251.3 ± 40.09	298.7 ± 23.35

*All results presented as mean ± SEM, with no significant differences (p ¿ 0.05) between Top and Bottom DA neurons. The “All neurons” column includes neurons localized to the top and bottom of the GL and those not initially distinguished by their glomerular localization.*

There were no significant differences between Top and Bottom neurons in regard to their membrane resistance (*n* = 64 neurons, *t*(62) = 0.8151, *p* = 0.4181, Figure 2Ci), capacitance (*n* = 64 neurons, *t*(62) = 1.050, *p* = 0.2979, Figure 2Di), neuronal areas (*n* = 64 neurons, *t*(62) = 0.1.050, *p* = 0.2979, Figure 2Ei), time constants (*n* = 64 neurons, *t*(62) = 0.82, *p* = 0.4153, Figure 2Fi), and action potential thresholds (*n* = 20 neurons, *t*(18) = 1.398, *p* = 0.1792, Figure 2Gi). Additionally, we observed a wide distribution of neuronal areas for these DA neurons (Figure 2H), which coincides with previous findings that OB DA neuron subtypes may be distinguished by their size (Pignatelli et al., 2005; Kosaka and Kosaka, 2009; Chand et al., 2015; Pignatelli and Belluzzi, 2017; Galliano et al., 2018; Kosaka et al., 2019).

Given the previous findings (Halász et al., 1981; Pignatelli et al., 2005; Kosaka and Kosaka, 2007, 2008, 2009) of differences in soma sizes between two potential populations of OB DA neurons and our finding that neuronal areas of DA neurons are widely distributed (Figure 2H), we also compared these same properties between recorded DA neurons with different sizes (Table 2). For these results, we compared neurons classified as “Large” (3390 to 4890 μm^2^, *n* = 24, green neurons) and with neurons classified as “Small” (1589 to 2610 μm^2^, *n* = 24, pink neurons) (Figure 2B). These separations are also marked by dashed lines, with each color corresponding to neuronal size (Figure 2H). The neuronal areas in these groups were significantly different from each other (*n* = 48 neurons, *t*(46) = 16.23, *p* < 0.0001, Figure 2Eii).

**TABLE 2:** Membrane properties of recorded OB DA neurons based on neuronal area.

	Large neurons	Small neurons
Membrane resistance (MΩ)	1274 ± 107.9	2401 ± 242.1***
Membrane capacitance (pF)	39.32 ± 0.917****	21.23 ± 0.633
Neuronal area (μm^2^)	3932 ± 91.73****	2123 ± 63.27
Time constant (ms)	50.41 ± 4.462	51.50 ± 5.549
Action potential threshold (pA)	417.8 ± 23.99****	181.7 ± 33.51

All data presented as mean ± SEM. ***p < 0.001; ****p < 0.0001.

The membrane resistance was significantly greater in Small neurons (*n* = 48 neurons, *t*(46) = 4.251, *p* = 0.0001, Figure 2Cii), and the capacitance was significantly greater in Large neurons (*n* = 48 neurons, *t*(46) = 16.23, *p* < 0.0001, Figure 2Dii). These results were expected, because membrane resistance and capacitance are a function of neuronal size (further functional implications are also addressed in the Discussion). There was no significant difference in time constants between Large and Small neurons (*n* = 48 neurons, *t*(46) = 0.1536, *p* = 0.8786, Figure 2Fii). The action potential thresholds were significantly greater in Large than Small neurons (*n* = 14 neurons, *t*(12) = 5.898, *p* < 0.0001, Figure 2Gii). These results are summarized in Table 2.

### General Action Potential Spiking Properties

**FIGURE 3:**
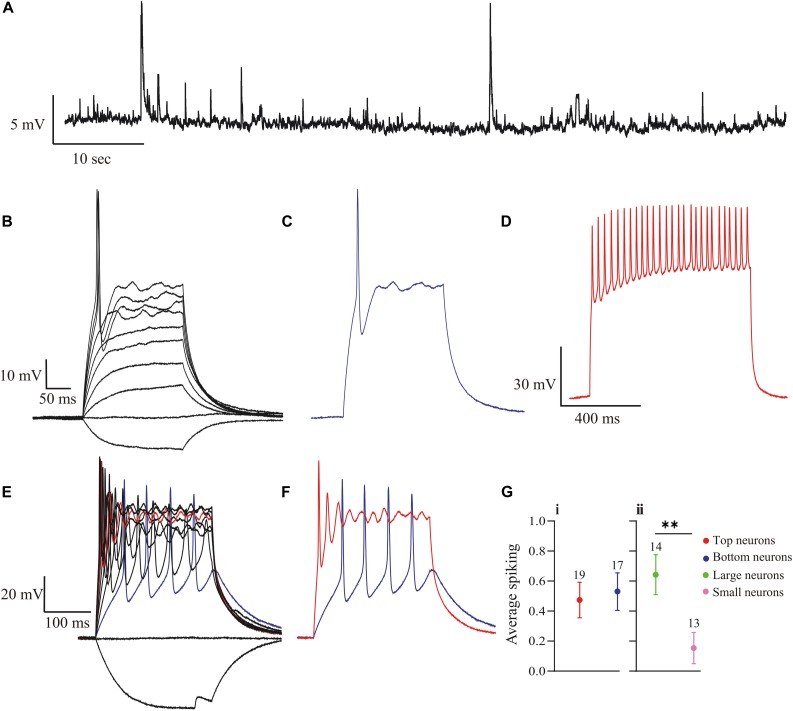
Action potential spiking properties of OB DA neurons. **(A)** There was no recorded spontaneous action potential activity (generated without stimulus input) in rat OB DA neurons. This recording shows synaptic activity, represented by EPSPs. **(B)** DA neurons fire a single action potential when stimulated with a sufficiently large depolarizing current. After firing an action potential, they go into depolarization block for the duration of the stimulus. These recordings resulted from incremental 10-pA steps, ranging from –10 to 80-pA. **(C)** A single trace from Figure 3B, which shows a single action potential generated from an 80-pA stimulus. **(D)** An example of a trace from a mitral cell (red trace), showing tonic firing in response to a 200-pA stimulus. **(E)** Some OB DA neurons fire multiple spikes when stimulated with a weaker stimulus (blue trace), but tend to fire a single spike with increasing stimulus strength (red trace). Each voltage trace is a response to incremental 25 pA stimuli, from –25 to 200 pA. **(F)** Example traces from Figure 3E, which show that a weak stimulus (25 pA in this example, blue trace) produced tonic action potential spiking, while a stronger stimulus (150 pA in this example, red trace) produced decaying spikes followed by a depolarization block. **(G)** To gauge if DA neurons have different spiking activity based on their glomerular localization and/or neuronal area, dummy variables were assigned to each spiking neuron (0 = no more than one spike at any depolarizing stimulus; 1 = multiple spiker at lower depolarizing stimuli only). There was no significant difference in average number of spikes between neurons based on their glomerular localization (*n* = 36, *p* = 0.7472, **Gi**), but there was a significant difference (*n* = 27, *p* = 0.0083**, **Gii**) between neurons based on neuronal area. Data represented as mean ± SEM.

All electrophysiology recordings were made in whole-cell current-clamp or voltage-clamp mode. The DA neurons were easily targeted for recording based on their green fluorescence (Figure 1). A total of 140 DA neurons were recorded in horizontal OB slices. The first set of experiments examined whether these neurons display spontaneous, non-synaptically driven action potential firing, which is a well-characterized property of mouse OB DA neurons (Pignatelli et al., 2005; Puopolo et al., 2005; Chand et al., 2015). None of the neurons examined (*n* = 32) fired spontaneous action potentials (Figure 3A). Many recordings showed evidence of EPSPs (the peaks in Figure 3A), indicating the presence of excitatory input to DA neurons, likely from OSNs, M/TCs, and/or ETCs, which may show that these neurons have reached maturity (Pignatelli et al., 2009). Some DA neurons did fire action potentials without stimulation (data not shown); however, these neurons appeared to be synaptically driven, because they did not show firing at a consistent frequency (4−12 Hz) associated with the spontaneous activity of OB DA neurons in mouse OBs (Pignatelli et al., 2005; Puopolo et al., 2005). Therefore, the firing activity of rat OB DA neurons cannot be considered spontaneous, representing a possible functional species difference between rat and mouse OBs.

We further examined the spiking profile of these neurons in response to depolarizing current step stimuli. Of the 60 DA neurons recorded for this activity, 59 neurons displayed single spiking activity at some stimulus level: in response to a sufficiently large depolarizing stimulus, these neurons produced a single action potential, followed by a plateau phase (depolarization block) for the duration of the stimulus (Figure 3B). While these neurons would display single spiking activity for some stimuli as low as 80 pA (Figure 3C). In contrast, mitral cells would display tonic spiking in response to a much larger 200 pA stimulus (Figure 3D). Thus, the DA neurons may be single spikers. However, of these 59 neurons, 27 neurons additionally produced multiple action potentials in response to weaker depolarizing stimuli (Figures 3E,F). As shown in Figure 3F, the multiple spike pattern produced with a weak stimulus (blue) was replaced by decaying spikes and depolarization block at a larger stimulus level (red). With even larger stimuli the cell becomes a single spiker. Therefore, these single spiker neurons are most responsive to weaker stimuli, so that they may act as high-pass filters (Korshunov et al., 2017; also see section “Discussion”).

Further, to analyze whether these spiking properties differ between Top and Bottom and/or Large and Small neurons, we assigned “dummy variables” to add a quantitative measure to these qualitative properties (0 = no more than one spike at any depolarizing stimulus; 1 = multiple spiking only at weaker depolarizing stimuli). After summating these values and comparing the means, there was no significant difference between the spiking properties of DA neurons based on glomerular localization (Top: 0.474 ± 0.117, *n* = 19; Bottom: 0.529 ± 0.125, *n* = 17; *n* = 36 neurons, *t*(34) = 0.3249, *p* = 0.7472, Figure 3Gi), but there was a difference based on the neuronal area of the neuron (Large: 0.6429 ± 0.133, *n* = 14; Small: 0.154 ± 0.104, *n* = 13; *n* = 27, *t*(25) = 2.866, *p* = 0.0083, Figure 3Gii). Therefore, larger DA neurons are more likely to produce multiple spikes in response to weaker depolarizing stimuli than are smaller DA neurons.

### Na^+^ Current

**FIGURE 4:**
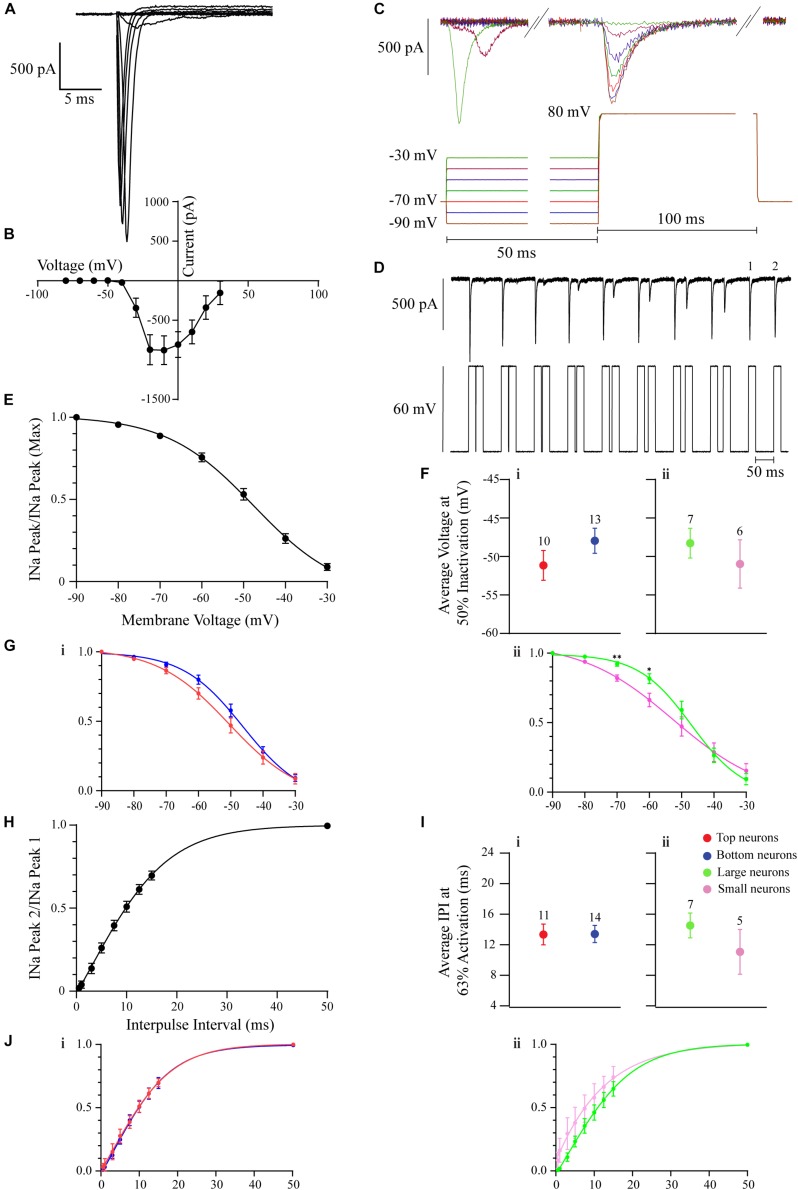
The voltage-gated I_Na_ in OB DA neurons. All recordings were performed in the presence of Cs and Cd. **(A)** Example of a group of I_Na_ from a DA neuron. These currents were activated by progressively depolarizing 200-ms 10 mV voltage steps, from –10 to 80 mV. Capacitance artifacts were manually blanked. **(B)** The current-voltage relationship (derived from 11 neurons) showing peaks of I_Na_. The largest peak amplitudes were produced when the membrane was depolarized between –20 and 0 mV. **(C)** An example of the protocol used to derive the inactivation/h∞ curve in panel **(E)**. 50 ms pre-pulse voltage steps ranged from –90 to –30 mV in 10 mV steps. Test 100 ms test pulse was 80 mV. Each color of the protocol trace is coordinated with the color of the current trace. **(D)** An example of the protocol used to derive the removal of inactivation/IPI curve in H. Neurons received paired voltage steps, depolarizing the membrane to 60 mV, with increasing subsequent IPIs (0.5, 1, 3, 5, 7.5, 10, 12.5, 15, and 50 ms). **(E)** The I_Na_ h∞ inactivation curve (derived from 23 neurons). Half of I_Na_ is inactive when the membrane is depolarized to –49 mV. **(F)** To gauge if I_Na_ inactivation properties differ between DA neurons based on their glomerular localization and/or neuronal area, their membrane voltages at 50% inactivation were compared. There was no significant difference between neurons based on localization (*n* = 23, *p* = 0.2149, **Fi**) or area (*n* = 13, *p* = 0.4645, **Fii**). **(G)** Inactivation curves were also compared between Top and Bottom **(Gi)** and Large and Small **(Gii)** neurons. For the membrane potentials of –70 and –60 mV, there were no significant differences between Top and Bottom neurons (–70 mV: *n* = 23, *p* = 0.1500; –60 mV: *n* = 23, *p* = 0.067), while there were significant differences between Large and Small neurons (–70 mV: *n* = 13, *p* = 0.0032**; –60 mV: *n* = 13, *p* = 0.0258*). **(H)** The I_Na_ IPI curve (derived from 25 neurons). Currents were activated with two 60-mV, 20-ms depolarizing steps. The activation time constant (τ = 63% of the channels are activated) is 13 ms. **(I)** To gauge if I_Na_ reactivation properties differ between DA neurons based on their glomerular localization and/or neuronal area, the average τ were compared. There was no significant difference between neurons based on localization (*n* = 25, *p* = 0.9710, **Ii**) or area (*n* = 12, *p* = 0.2913, **Iii**). **(J)** Individual IPI curves were also constructed for Top and Bottom **(Ji)** and Large and Small **(Jii)** DA neurons. These two sets of curves were similar. Data points are represented as mean ± SEM.

In whole-cell voltage-clamp, all recorded neurons displayed the fast-activating and inactivating inward I_Na_ (Figure 4A). To isolate the I_Na_ in these neurons, recordings were performed with a 132 mM Cs-based intracellular solution and bath-applied 100 μM Cd (see section “Materials and Methods”). The transient fast activating and inactivating I_Na_ was often no longer than 10 ms (Figure 4A). Recordings from 33 neurons were made for these experiments. The I_Na_ current-voltage curve shows that peak current is largest when the membrane is depolarized to between −20 and 0 mV (*n* = 11 neurons; Figure 4B).

To visualize the inactivation properties of this current, a I_Na_ h_∞_-curve (Figure 4E, but also see section “Materials and Methods”) was derived. An example protocol used to derive this curve is included in Figure 4C. The resulting I_Na_ inactivation curve shows half-inactivation at −49 mV (*n* = 23 neurons; Figure 4E). To determine if these I_Na_ inactivation properties differ between DA neurons based on their localization or neuronal area, an inactivation curve was derived for each neuron, and the individual membrane potentials at 50% I_Na_ inactivation were summated and their means compared. There were no significant differences based on glomerular localization (*n* = 23 neurons, *t*(21) = 1.279, *p* = 0.2149, Figure 4Fi) or neuronal area (*n* = 13 neurons, *t*(11) = 0.7577, *p* = 0.4654, Figure 4Fii) of OB DA neurons (Table 3). To further determine if this current contributes to the spiking difference seen in Figure 3Gii, we examined and compared the inactivation curves of Top and Bottom (Figure 4Gi) and Large and Small (Figure 4Gii) neurons at membrane potentials of interest (−70 and −60 mV). This comparison was prompted by our observation that spiking differences between Large and Small neurons with weaker current stimuli (closer to their resting potential) would disappear when those stimuli increased in strength. Whereas the inactivation curve for Top neurons was left shifted from that for the Bottom neurons, there were no significant differences between the Top and Bottom inactivation curves at either of the membrane potentials of interest (−70 mV: *n* = 23 neurons, *t*(21) = 1.494, *p* = 0.15; −60 mV: *n* = 23 neurons, *t*(21) = 1.926, *p* = 0.0677). Similarly, the inactivation curve for Small neurons was left shifted from that of Large neurons, and there were significant differences at both membrane potentials (−70 mV: *n* = 13 neurons, *t*(11) = 3.748, *p* = 0.0032; −60 mV: *n* = 13 neurons, *t*(11) = 2.576, *p* = 0.0258) between Large and Small neurons’ inactivation curves. This finding suggests that Small DA neurons’ Na_v_s are more sensitive to inactivation that Large neurons at membrane potentials close to the resting membrane potential.

**TABLE 3:** I_Na_ properties of OB DA neurons.

	Top	Bottom	Large	Small	All
50% I_Na_ inactivation (mV)	−51.16 ± 1.93	−47.59 ± 1.62	−48.27 ± 1.934	−57.16 ± 3.14	−49
IPI (ms)	13.35 ± 1.353	13.41 ± 1.128	14.53 ± 1.633	11.07 ± 2.918	13

*All data, except for the “All” category presented as mean ± SEM. There were no significant differences between Top and Bottom DA neurons or between Large and Small DA neurons.*

Lastly, to characterize the rate of recovery from inactivation, an IPI I_Na_ curve (Figure 4H, but also see section “Materials and Methods”) was derived. An example protocol used to derive this curve is included in Figure 4D. The resulting IPI curve shows that the average time that it takes for 63% of Na_v_ channels to recover from inactivation (τ) was 13 ms (*n* = 25 neurons; Figure 4H). Again, to determine if these properties differed between DA neurons based on their localization or neuronal area, an IPI curve was derived for each neuron, and the individual τ values were summated and their means compared. There were no significant differences based on glomerular localization (*n* = 25 neurons, *t*(23) = 0.03674, *p* = 0.9710, Figure 4Ii) or neuronal area (*n* = 12 neurons, *t*(10) = 1.114, *p* = 0.2913, Figure 4Iii) of these DA neurons (Table 3). To further determine if recovery from Na_v_ channel inactivation influences the spiking difference seen in Figure 3Gii, we constructed and compared IPI curves for Top and Bottom (Figure 4Ji) and Large and Small (Figure 4Jii) neurons. These curves were similar between Top and Bottom and Large and Small neurons throughout increasing IPIs.

### H-Current

**FIGURE 5:**
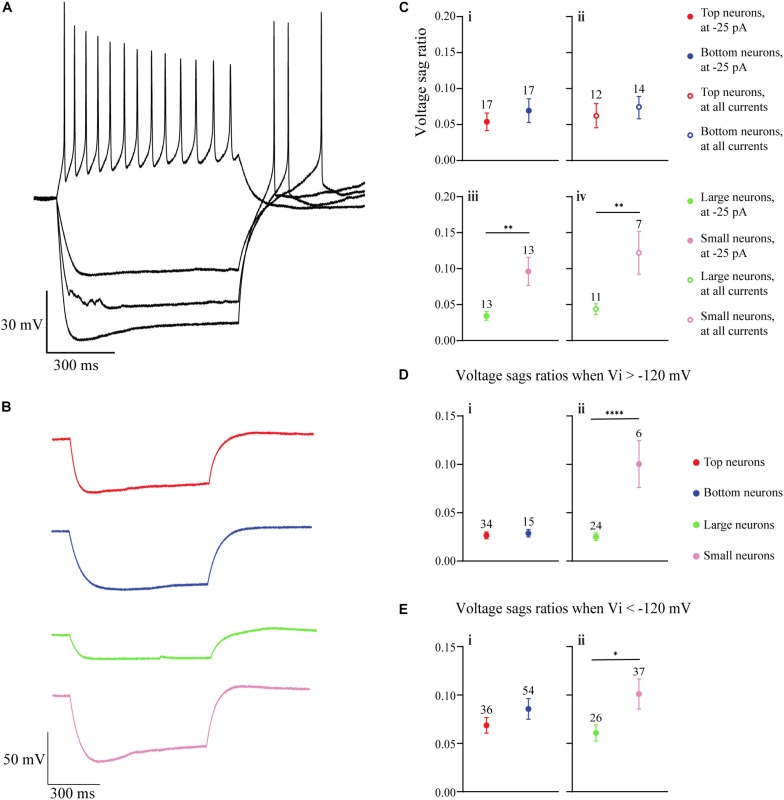
The hyperpolarization-activated, non-specific cation I_H_ in OB DA neurons is identified by upward voltage sags during hyperpolarization, after hyperpolarization depolarization, and (sometimes) an action potential following hyperpolarizing stimuli. **(A)** An example of a DA neuron showcasing these three properties of I_H_ during three hyperpolarizing current (–25, –50, and –75 pA) injections. **(B)** Representative hyperpolarizing traces of each of the four groups of DA neurons from this study (red = Top, blue = Bottom, green = Large, pink = Small). All traces are scaled to the scale on the bottom left of the figure. **(C)** A comparison of voltage sag ratios of DA neurons based on their glomerular localization and neuronal area. There was no significant difference between DA neurons based on their glomerular localization, either when the neurons received a –25-pA stimulus (“at –25 pA,” *n* = 34, *p* = 0.4500, **Ci**) or when receiving a combination of –25, –50, and –75 pA, or all three hyperpolarzing currents (“at all currents,” *n* = 26, *p* = 0.5904, **Cii**). There were significant differences between DA neurons based on the neuronal area, both when receiving only a –25-pA stimulus (*n* = 26, *p* = 0.0061**, **Ciii**) and when receiving the combination of hyperpolarizing currents (*n* = 18, *p* = 0.0067**, **Civ**). **(D)** Voltage sag ratios of DA neurons were compared at starting membrane potentials positive to –120 mV. There was no significant difference between neurons based on glomerular localization (*n* = 49 sags, *p* = 0.6784, **Di**), but Small DA neurons had a significantly greater voltage sag ratio than Large neurons (*n* = 30 sags, *p* < 0.0001****, **Dii**). **(E).** Voltage sag ratios of DA neurons were also compared at membrane potentials negative to –120 mV. Again, there was no significant difference between neurons based on glomerular localization (*n* = 90 sags, *p* = 0.2576, **Ei**), but Small DA neurons had a significantly greater voltage sag ratio than Large neurons (*n* = 63 sags, *p* = 0.0432*, **Eii**). All data represented as mean ± SEM.

The I_H_ (a non-specific cation current activated during hyperpolarization) is produced by the hyperpolarization-activated cyclic nucleotide-gated (HCN) channels (Biel et al., 2009; Wahl-Schott and Biel, 2009). Because the I_H_ has been shown to indirectly influence the resting membrane potential of OB DA neurons (Pignatelli et al., 2013), and because it influences several spiking frequencies in the hippocampus (Biel et al., 2009), we analyzed its strength as a possible metric that could contribute to spiking differences between Large and Small DA OB neurons (Figure 3Gii). The presence of I_H_ in our recordings was identified by depolarizing voltage sags in the membrane potential when a sufficiently large hyperpolarizing current is applied. It can also contribute to rebound spiking after the removal of the hyperpolarizing applied current. Both sag and rebound spiking are exemplified in Figure 5A, confirming that rat OB DA neurons possess I_H_. Examples of individual hyperpolarizing traces and the presence of voltage sags are also shown for Top, Bottom, Large, and Small DA neurons (Figure 5B).

The voltage sag ratio (see section “Materials and Methods” for calculation methods), a proxy of the slowly activating I_H_ (see section “Discussion”), was used to differentiate between OB DA neurons based on their localization in the GL and neuronal areas. Two sets of voltage sag ratios were used: one set from voltage traces resulting from a −25-pA stimulus only and the second from traces resulting from some combination of −25, −50, or −75 pA stimuli (or all three). For voltage sags resulting from a −25-pA stimulus only, there was no significant difference between the voltage sag ratios of DA neurons based on glomerular localization (*n* = 34 neurons, *t*(32) = 0.765, *p* = 0.45, Figure 5Ci), but there was a significant difference based on neuronal area (*n* = 26 neurons, *t*(24) = 3.009, *p* = 0.0061, Figure 5Ciii; Table 4). Likewise, for voltage sags resulting from the combination of hyperpolarizing stimuli (“all currents” in the figure legend), there was no significant difference between the voltage sag ratios of DA neurons based on localization (*n* = 26 neurons, *t*(24) = 0.5456, *p* = 0.5904, Figure 5Cii), but there was a significant difference based on neuronal area (*n* = 18 neurons, *t*(16) = 3.113, *p* = 0.0067, Figure 5Civ; Table 4).

**TABLE 4:** I_H_ properties of OB DA neurons.

	Top	Bottom	Large	Small
Voltage sag ratio (−25 pA)	0.05 ± 0.01	0.07 ± 0.02	0.03 ± 0.01	0.10 ± 0.05**
Voltage sag ratio (all)	0.06 ± 0.02	0.07 ± 0.02	0.04 ± 0.01	0.12 ± 0.03**
V_i_ positive to −120 mV	0.03 ± 0.2×10^−2^	0.03 ± 0.3×10^−2^	0.03 ± 0.3×10^−2^	0.10 ± 0.02****
V_i_ negative to −120 mV	0.07 ± 0.8×10^−2^	0.09 ± 0.01	0.06 ± 0.8×10^−2^	0.10 ± 0.02*

All data presented as mean ± SEM. There were no significant differences between Top and Bottom DA neurons, but there were several significant differences between Large and Small DA neurons. *p < 0.05; **p < 0.01; ****p < 0.0001.

A reason why smaller DA neurons display larger voltage sag ratios, possibly indicating a stronger overall I_H_, is due to the large resistance of these neurons. However, Small DA neurons may also possess a larger HCN channel density than Large neurons, which would further contribute to a larger voltage sag ratio in the Small population. To test this hypothesis, we divided the voltage sag ratios into two groups based upon the voltage that they dropped to immediately upon application of the hyperpolarizing applied current (V_i_). In one group, the V_i_ was greater than (positive to) −120 mV, and in the second group V_i_ was less than (negative to) −120 mV. This division was made since HCN channels are typically almost entirely activated at potentials below −120 mV (Ross et al., 2017), so this second group should have almost maximally activated channels. If Small neurons possess greater voltage sag ratios than Large neurons at these potentials, then they will likely have a stronger I_H_, possibly due to a greater density of HCN channels. When the V_i_ was positive to −120 mV, the voltage sag ratio did not significantly differ between DA neurons based on their glomerular localization (*n* = 49 sags, *t*(47) = 0.4173, *p* = 0.6784, Figure 5Di) but the voltage sag ratio was significantly greater in Small versus Large neurons (*n* = 30 sags, *t*(28) = 5.547, *p* < 0.0001, Figure 5Dii; Table 4). When the V_i_ was negative to −120 mV, there was again no significant difference in the voltage sag ratio between DA neurons based on their glomerular localization (*n* = 90 sags, *t*(88) = 1.139, *p* = 0.2576, Figure 5Ei), but the voltage sag ratio was significantly greater in Small versus Large neurons (*n* = 63 sags, *t*(61) = 2.064, *p* = 0.0432, Figure 5Eii; Table 4). Thus, Small DA neurons possessed a stronger I_H_ than Large DA neurons, regardless of how the sag ratio quantification was performed.

### Current Ramps Reveal Spiking Properties

**FIGURE 6:**
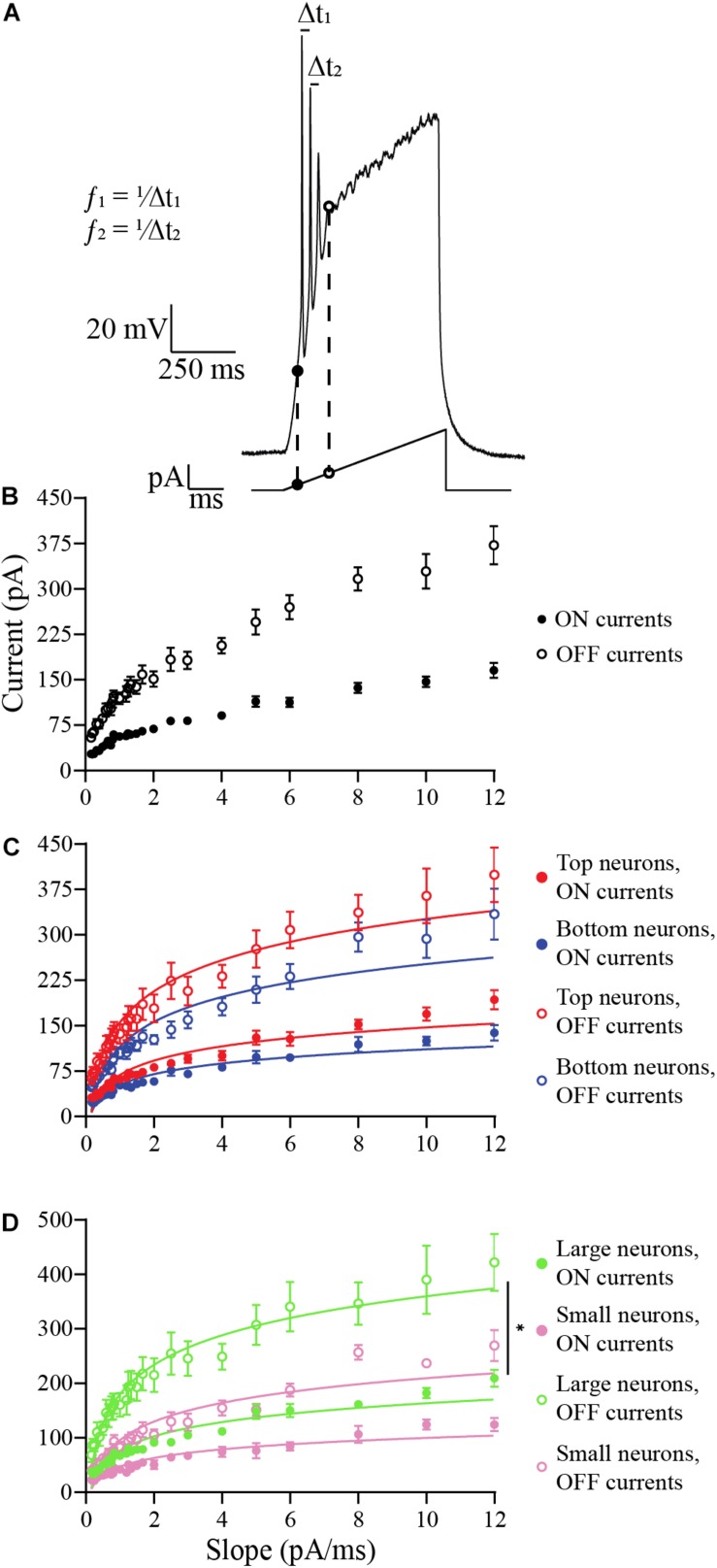
Analysis of the effects of ramp slopes (in pA/ms) on the ON and OFF currents of OB DA neurons. **(A)** Example of a ramp protocol (bottom) and a resulting voltage trace (top). The traces and their corresponding ramps were used to determine the ON (black circles) and OFF (white circles) currents. This figure also shows an example of how spike frequencies are derived from traces of ramp protocols (data shown in Figure 7). Of the 42 ramps used, some ramps had identical slopes (e.g., the slope of 2 pA/ms can include ramps of 200 pA for 50 ms, 400 pA for 100 ms, etc.). For different ramp protocols with the same slopes, the ON and OFF currents and the spike frequencies and number of action potentials in the next figure were averaged. **(B)** Averaged ON and OFF current responses to ramp slopes (derived from 19 neurons). Increasing the slope increases the ON and OFF current of all neurons, but the largest effect is on the OFF current. This also indicates a decrease in duration of spiking with increasing ramp slopes. **(C)** A comparison of ON and OFF currents of DA neurons based on their glomerular localization. After transforming these power functions into log-log plots (see section “Materials and Methods”), there was no significant difference between the increasing ON (*n* = 19, *p* = 0.3405) and OFF currents (*n* = 19, *p* = 0.5368) with increasing ramp slopes between Top and Bottom neurons. **(D)** Same comparison between DA neurons based on their neuronal areas. There were no significant differences in the increasing ON current (*n* = 10, *p* = 0.7680) with increasing ramp slopes, but there was a difference in the increasing OFF currents (*n* = 10, *p* = 0.0402*) between Large and Small neurons. All data represented as mean ± SEM.

In the next experiments, we applied ramp stimulus protocols (Figure 6A), where the input current is gradually increased to a peak and is then removed. This protocol was used to avoid or postpone the depolarization block that occurs in OB DA neurons when the input current is applied as a step pulse (Figure 3), allowing analysis of spiking properties of the neurons. The ramp protocols used for the following experiments varied in 6 amplitudes (starting with 0 pA and increasing to either 100, 200, 300, 400, 500, or 600 pA) over 7 durations (50, 100, 200, 300, 400, 500, or 600 ms). In total, 42 ramps were used. The ramps with longer duration and smaller current amplitude have shallow slopes (in pA/ms), so there is a more gradual application of the stimulus, and the ramps with shorter duration and large current amplitudes have steep slopes.

In the first experiment, we examined how the ramp slopes influenced the amount of current required for a neuron to begin spiking (“ON current”) and end spiking (“OFF current”) (Figure 6A). At the OFF current, a depolarization block is initiated that lasts for the duration of the stimulus. A total of 19 DA neurons were tested, including neurons that produced one or more action potential spike(s) per ramp. Steeper ramp slopes consistently resulted in larger ON and OFF currents in all DA neurons tested (Figure 6B). This result indicates that neurons fire over a longer range of current when stimulated with steep slopes, but their duration of firing decreases with increasing ramp slopes ($\text{Duration}=\left(\frac{\mathrm{OFF}\,\mathrm{current}}{\mathrm{Ramp}\,\mathrm{slope}}\right)-\left(\frac{\mathrm{ON}\,\mathrm{current}}{\mathrm{Ramp}\,\mathrm{slope}}\right)$). The changes in these responses were then compared between DA neurons based on GL localization and neuronal area.

The data appear to be distributed as power functions, *y* = 10^*b*^⋅x^*m*^, where *b* and *m* are parameters. For this reason, we transformed the data by taking the common logarithm of the x (ramp slope) and *y*-values (ON- or OFF-current, or spike frequency, or number of spikes) and constructing log-log plots (see section “Materials and Methods” and also Supplementary Figures). This linearized the data, confirming the power-law dependence of the data on the ramp slope, and we looked for significant differences in the slopes *m* of the linearized data (this parameter is the exponent of the power function). There were no significant difference in *m* between Top and Bottom neurons in their increasing ON currents (Top: *b* = 1.767, *m* = 0.4494, *n* = 9 neurons; Bottom: *b* = 1.657, *m* = 0.4281, *n* = 10 neurons; *p* = 0.3405, Supplementary Figure 1A) nor in their increasing OFF currents (Top: *b* = 2.116, *m* = 0.4359, *n* = 9 neurons; Bottom: *b* = 1.993, *m* = 0.4537, *n* = 10 neurons; *p* = 0.5368, Supplementary Figure 2A) with increasing ramp slopes. The raw, un-transformed data are shown in Figure 6C. For the Large and Small DA neurons, there was no difference in *m* between their increasing ON currents with increasing ramp slope (Large: *b* = 1.827, *m* = 0.4274, *n* = 5 neurons; Small: *b* = 1.612, *m* = 0.4210, *n* = 5 neurons; *p* = 0.7680, Supplementary Figure 1B), but there was a significant difference in *m* between the increasing OFF currents of Small and Large DA neurons (Large: *b* = 2.194, *m* = 0.4026, Small: *b* = 1.905, *m* = 0.4729; *p* = 0.0402, Supplementary Figure 2B). The un-transformed data are shown in Figure 6D. These results indicate that the OFF current for Small neurons increases significantly more with increases in the current ramp slope than does the OFF current for Large neurons, however, Large neurons still have larger OFF currents when stimulated with this range of ramp stimuli (see Supplementary Figure 2B and section “Discussion”). There are no significant differences in either ON or OFF currents between Top and Bottom neurons.

**FIGURE 7:**
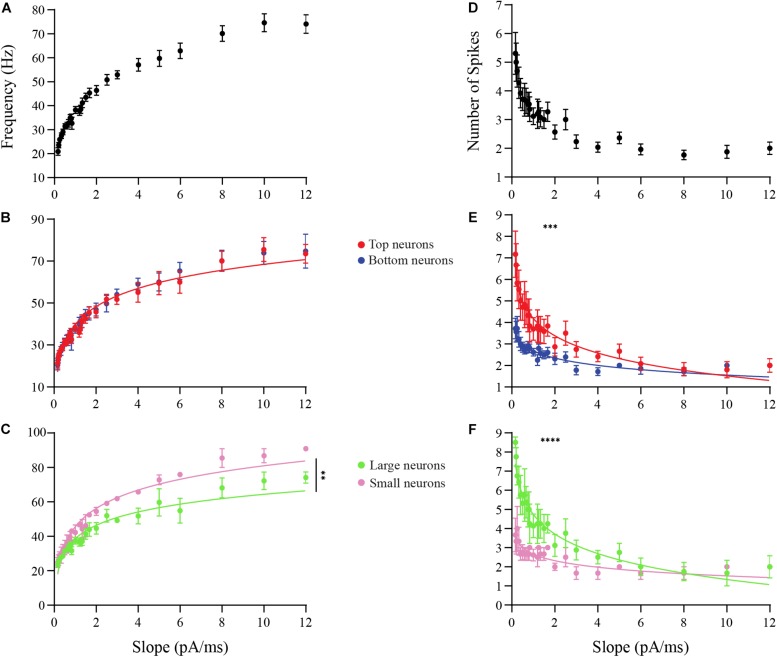
Analysis of the effects of ramp slopes on the spike frequencies and the number of spikes of OB DA neurons. **(A)** The frequency responses (in Hz) of neurons (*n* = 13) that spiked more than once per ramp stimulus increase with increasing ramp slopes. **(B)** After transforming these power functions into log-log plots (see section “Materials and Methods”), there was no difference (*n* = 13, *p* = 0.3544) in the increasing spike frequencies with increasing ramp slopes between Top and Bottom DA neurons. **(C)** The increasing spike frequencies with increasing ramp slopes was significantly higher (*n* = 7, *p* < 0.0043**) in Small than Large neurons. **(D)** Number of spikes produced with increasing ramp slopes of neurons (*n* = 13) drops dramatically, particularly between 0 and 2 pA/ms ramps. **(E)** Top DA neurons produced a significantly greater decrease in spikes across increasing ramp slopes than did Bottom neurons (*n* = 13, *p* = 0.0009***). **(F)** There was an even greater decrease in spikes across increasing ramp slopes in Large compared to Small DA neurons (*n* = 7, *p* < 0.0001****).

Next, we examined the frequency response of OB DA neurons over a range of input ramp slopes. Spike frequency was calculated for each individual action potential by measuring the time period (Δt) between that action potential and the next one, and taking the reciprocal of the period to determine frequency (*f*) (Figure 6A). This was then averaged over all spikes in the response. A total of 13 neurons that produced more than one action potential per ramp were used for these experiments. Overall, the spike frequency increased with increasing ramp slopes, up to a saturation frequency (∼70−75 Hz) (Figure 7A). The change in spike frequency was used as another metric to compare DA neurons based on glomerular localization and neuronal area. The *m* for the increase in spike frequency across ramp stimuli did not differ between Top and Bottom DA neurons (Top: *b* = 1.568, *m* = 0.2900, *n* = 6 neurons; Bottom: *b* = 1.567, *m* = 0.3068, *n* = 7 neurons; *p* = 0.35, Supplementary Figure 3A). The un-transformed data are shown in Figure 7B. However, the significantly higher *m* in Small neurons indicates that they had a greater increase in spike frequency than Large neurons across increasing ramp slopes (Large: *b* = 1.566, *m* = 0.2649, *n* = 4 neurons; Small: *b* = 1.626, *m* = 0.3217, *n* = 3 neurons; *p* = 0.004, Supplementary Figure 3B). The un-transformed data are shown in Figure 7C. Thus, Small DA neurons appear to increase their spike frequency with increasing ramp slope strength more than the DA Large neurons.

Lastly, we determined the effect of ramp slope on the number of action potentials produced by these neurons. Again, 13 neurons with multiple action potentials per ramp stimulus were used. Overall, the number of action potential spikes decreased with increasing ramp slopes (Figure 7D). We observed differences in this change in the number of action potentials between DA neurons based on both GL localization and neuronal area. Interestingly, Top DA neurons had a significantly more negative *m* than Bottom neurons, and thus produced a greater decrease in spikes across increasing ramp stimuli than Bottom neurons (Top: *b* = 0.5636, *m* = −0.3035, *n* = 6 neurons; Bottom: *b* = 0.4046, *m* = −0.1875, *n* = 7 neurons; *p* < 0.001, Supplementary Figure 4A). The un-transformed data are shown in Figure 7E. The *m* value was even more significantly negative in Large neurons, indicating that they had an even greater decrease in spikes across increasing ramp stimuli than Small DA neurons (Large: *b* = 0.6208, *m* = −0.3859, *n* = 4 neurons; Small: *b* = 0.3994, *m* = −0.1909, *n* = 3 neurons; *p* < 0.0001, Supplementary Figure 4B). The un-transformed data are shown in Figure 7F.

These data suggest that slowly increasing inputs result in long, low-frequency responses, while inputs that increase rapidly result in short, high-frequency responses. The number of spikes produced during a current ramp declines faster with the ramp slope in the Top DA neurons than in the Bottom DA neurons. The greatest difference in the number of spikes between these groups appears at shallower ramps (0−7 pA/ms). The Small DA neurons exhibit a greater increase in spike frequency with increase in the current ramp slope than do the Large DA neurons. These same Small DA neurons exhibit less of a decrease in the number of spikes produced as current ramp slope is increased than do the Large DA neurons. We note that the ramp protocol was the only protocol that we applied that was able to distinguish some differences between Top and Bottom DA neurons.

## Discussion

We used a novel transgenic rat line (TH-GFP) to show that DA neurons are widely expressed in the GL of the rat OB. The interneurons that express TH and GABA are collectively termed JGCs, which are among the first neurons to make contact with the OSNs. In the GL, DA, GABA, and glutamate can modulate the odor signal being transmitted to the main output neurons, the M/TCs. These modulatory mechanisms include inhibition of glutamate release from OSNs via presynaptic activation of the D_2_ and GABA_*B*_ receptors (Baker, 1986; Nickell et al., 1994; Hsia et al., 1999; Berkowicz and Trombley, 2000; Ennis et al., 2001; Vaaga et al., 2017), inhibition of glutamate release from M/TCs via D_2_ receptor activation (Davila et al., 2003), and an interglomerular inhibition-excitation of ETCs via activation of GABA_*A*_ and D_1_ receptors, respectively (Liu et al., 2013). While there is much understanding about the synaptic activity of OB DA neurons, it is not fully understood if there are more than one type of OB DA neuron and how these neurons respond to artificial stimuli. Based on our examination of membrane properties, we show that OB DA neurons may be differentiated according to their neuronal area, but not always according to their glomerular localization (whether closer to the ONL or the EPL) in the GL. While most membrane properties could not be differentiated between neurons based on their glomerular localization, responses to ramp stimuli, including the ON and OFF currents and the number of spikes as the ramp slope increased, differed between both Top and Bottom neurons and Large and Small neurons. The spiking profiles of these neurons in response to step stimuli were distinguishable by their neuronal area and sometimes by their glomerular localization. Along with these findings, we conclude by discussing potential species differences between OB DA neurons.

### Evidence for and Potential Identity of at Least Two Types of Olfactory Bulb Dopamine Neurons

Previous findings commonly categorize OB DA neurons into two size profiles (Halász et al., 1981; Pignatelli et al., 2005; Kosaka and Kosaka, 2007, 2008, 2009, 2011; Chand et al., 2015; Pignatelli and Belluzzi, 2017; Galliano et al., 2018; Kosaka et al., 2019). It is possible that OB DA neurons with smaller soma sizes that sometimes lack an axon are PGCs (Pinching and Powell, 1971; Kosaka and Kosaka, 2011; Nagayama et al., 2014), while DA neurons with larger soma sizes and interglomerular projections are SACs (Aungst et al., 2003; Kiyokage et al., 2010; Bywalez et al., 2017). Are these two potentially different populations of OB DA neurons differently localized in the GL? Our membrane properties results indicate that there is no preferred glomerular localization of Large and Small DA neurons. However, a recent study by Galliano et al. (2018) found that large TH + /DA neurons were mostly expressed in the deep glomerulus, at the border of GL/EPL. Interestingly, these large neurons exclusively possessed an AIS, indicating that these DA neurons are axonic (Galliano et al., 2018). While we have recorded a total of 10 Large DA neurons that were localized to the Bottom of the GL, we also recorded from a total of 15 Large DA neurons that were localized to the Top of the GL. Thus, our data suggest that Large DA neurons are found in both the superficial and deep halves of the glomerulus. While Large DA neurons in the deep GL likely possess an axon, Large neurons in the superficial GL may not possess an axon.

A new study by Kosaka et al. (2019) has further described the OB DA population as belonging to four groups: the Large PGCs with apparent axons, Small PGCs that are either axonic or anaxonic, Transglomerular cells with processes extending up to two or more glomeruli, and the Incrusting cells that extend their processes in the periphery of the glomeruli. These findings, along with our results on the many differences (passive membrane properties, tonic spiking or single action potentials, the I_H_, OFF currents, spiking frequency, and number of spikes produced) between the “Large” and “Small” OB DA neurons, support the previous findings that there are at least two types of OB DA neurons. It is also clear that, given that there are new ways of differentiating these neurons based on their dendritic arborizations (Bywalez et al., 2017) and projections of their processes (Kiyokage et al., 2010; Kosaka et al., 2019), further studies to better understand OB DA neurons should focus on their spiking properties.

### Spiking Properties, Ionic Currents, and Further Evidence for at Least Two Types of Dopamine Neurons in the Olfactory Bulb

When depolarized by conventional current step stimuli, OB DA neurons overwhelmingly (*n* = 59/60 neurons) produced a single action potential at the beginning of a strong depolarizing stimulus, before entering a depolarization block for the duration of the stimulus (Figures 3B,C). This was in contrast to the tonic spiking produced in mitral cells (Figure 3D). Some (*n* = 27/59 neurons) of these single spikers also produced multiple spikes continuously when stimulated with weaker stimuli only (Figures 3E,F). Therefore, we classified these neurons as single spikers that are more responsive to weaker stimuli. These neurons may fit the criteria of the “non-accommodating” spiking group characterized by McQuiston and Katz (2001), because when they produced tonic spiking, these spikes appeared to maintain a consistent spike frequency throughout the step stimulus. Based on the number of Large and Small neurons that showed these properties, it was more likely that Large OB DA neurons would produce multiple spikes at weaker stimuli, but not Small neurons (Figure 3G). The spiking data imply that the larger OB DA neurons are more responsive to weaker, not stronger, odor stimuli.

To examine the role that ionic currents play in these neurons being single spikers, we investigated the I_Na_ (Figure 4). Of these ionic properties, we found that Small DA neurons’ Na_v_ channels were much more sensitive to inactivation at membrane potentials close to the resting membrane potential (−70 and −60 mV) than those of Large DA neurons (Figure 4Gii). Thus, this difference in Na_v_ sensitivity should, at least partially, address the difference in spiking between Large and Small DA neurons (Figure 3Gii). Future studies that could address this difference in spiking could include investigating a potential difference in the density of Na_v_ between Large and Small DA neurons (Zengel et al., 1985; Sengupta et al., 2013), the neuronal localization of these channels (Trimmer and Rhodes, 2004; Kress and Mennerick, 2009), and further analyses of K^+^ currents, including the A-type (Iseppe et al., 2016) and M-currents (Nai et al., 2011; Li et al., 2015). Our reported time constant (13 ms, Figure 4H) is similar to the previously reported 16.8 ms in OB PGCs (Iseppe et al., 2016). In OB PGCs, it was determined that the long time constant required to remove inactivation from Na_v_ and the short time constant required to remove the inactivation from channels that produce the K^+^ A-current contribute to the single spiking properties of OB PGCs (Iseppe et al., 2016). Given our reported values for inactivation and the similarly long removal of inactivation time constant for Na_v_, these properties may contribute to the single spiking activity of OB DA neurons.

The I_H_ can act as a pacemaker current for neurons that experience spontaneous, rhythmic spiking (Wahl-Schott and Biel, 2009). In mouse OB DA neurons, pharmacological blockade of I_H_/HCN did hyperpolarize their resting membrane potential, but this did not cause these neurons to stop their spontaneous spiking (Pignatelli et al., 2013). We did not test the importance of the I_H_ in the firing properties of rat OB DA neurons. However, as the neurons in our study did not produce spontaneous spikes (Figure 3A), it is likely that this current does not act as a pacemaker in rat OB DA neurons either. We observed further biophysical difference between Large and Small OB DA neurons in the form of the I_H_. The presence of I_H_ in these neurons was evident, because they produced voltage sags when a hyperpolarizing current was applied, often followed by rebound action potentials that can be due in part to I_H_ (Figure 5A). We used the voltage sag ratio as a representative measure of the strength of I_H_ and as a means to distinguish between potential types of OB DA neurons. At all hyperpolarizing stimuli, voltage sag ratios did not differ between DA neurons based on GL localization, but were consistently larger in Small compared with Large DA neurons (Figures 5Ciii,iv,Dii,Eii). One functional implication of I_H_ could be that it allows for the smaller neurons to get out of hyperpolarization, bypass their action potential thresholds (which would be easier for these neurons since smaller neurons have a lower threshold, Figure 2Gii), and generate an action potential earlier than larger neurons. Given the inactivation properties of Na_v_s in Small neurons, their larger I_H_ can indirectly inactivate these channels more than it would in Large DA neurons, which may contribute to the difference in spiking between these neurons.

According to Ohm’s Law, smaller neurons should produce a greater voltage drop when hyperpolarized than larger neurons, activating a larger fraction of HCN channels. Thus, smaller neurons would be expected to produce larger voltage sags, as well. However, if larger DA neurons experienced the same voltage drop as smaller neurons, would their voltage sag ratios be different or the same? As we found that Small DA neurons experienced larger voltage sag ratios even when they began at similar membrane potentials as Large DA neurons (Figures 5D–E), we conclude that Small DA neurons have a stronger I_H_ than Large neurons. Interestingly, the difference in voltage sag ratios between Small and Large neurons was much greater at more positive hyperpolarized potentials (Figure 5D) than more negative hyperpolarized potentials (Figure 5E). This suggests that the HCN channel activation curve could be right shifted in the smaller neurons, so that the channels activate at higher voltages.

It should be noted that, while some of the recorded neurons did not have noticeable voltage sags, it does not necessarily mean that they do not possess an I_H_. Depending on the presence of specific HCN subunits (subunits 1−4; Wahl-Schott and Biel, 2009; Meredith et al., 2012), these neurons may possess the fast-activating I_H_, slow-activating I_H_, or a mixture of both. The fast-activating I_H_ rapidly opposes the applied hyperpolarizing current, reducing the size of the voltage drop when the hyperpolarizing current is applied. In contrast, the slow-activating I_H_ produces the voltage sags (Ross et al., 2017). Therefore, those neurons that did not display voltage sags (a property of slow-activating I_H_) may still possess the fast-activating I_H_. Future experiments could label the HCN subunits and verify the distribution of the fast and slow components of the I_H_ among different rat OB DA neurons, as has recently been done in vestibular ganglion neurons (Michel et al., 2015).

### Further Spiking Properties in Response to Current Ramp Stimulations

Our current clamp data up to this point show spiking in response to single step stimuli. While current step protocols provide a good snapshot of the spiking response per individual stimulus, we wanted to further characterize spiking properties in response to increasing stimuli. Thus, we used ramp stimuli, which can be thought of as a new current step stimulus every millisecond. Ramps with smaller current amplitudes and longer durations had shallow slopes, while ramps with larger amplitudes and shorter duration had steep slopes (“ramp slopes” is interchangeable with “ramp stimuli”). The resulting power functions (Figures 6, 7) and their transformed log-log plots (see section “Materials and Methods” and Supplementary Figures) describe the response of these neurons to increasing ramp stimuli, as well as differences between the responses of Top vs. Bottom and Large vs. Small DA neurons.

Shallow ramp stimuli yielded smaller ON/OFF currents, while steeper stimuli yielded larger ON/OFF currents (Figure 6B). Shallow ramp stimuli also yielded smaller spike frequencies (Figure 7A) and more spikes (Figure 7D) than steep stimuli. Large neurons produced larger OFF currents than Small neurons (Figure 6D). This is consistent with data in Figure 3Gii, because Large neurons would take a longer time to enter depolarization block than Small neurons, especially at very shallow ramp stimuli (Duration = Current/Ramp slope). Small neurons developed larger spike frequencies across increasing ramp stimuli (Figure 7C) and had considerably fewer spikes at shallow ramp stimuli (Figure 7F) than Large neurons. This again confirms our findings that not only are OB DA neurons more sensitive to weaker stimuli, but Large DA neurons tend to develop more, lower interspike frequency action potentials than Small neurons. While these results confirm our hypothesis for Large and Small neurons based on Figure 3, the findings between Top and Bottom neurons are less intuitive.

There are two parameters – derived from log-log plots – that influence these DA neurons: 10*^*b*^* and x*^*m*^*. Whereas 10*^*b*^* is a constant, x*^*m*^* changes with increasing ramp stimuli. If the exponent *m* (which is the slope of the linear equations generated in log-log plots, and also the exponent of the ramp slope stimulus in the un-transformed power functions) is significantly greater in one group, then the change that group experiences will increase (or decrease, if *m* is negative) more than the other group. Small neurons experience a greater increase in their spike frequencies (Supplementary Figure 3B), with a smaller decrease in their overall spiking (Supplementary Figure 4B), compared to Large neurons as ramp stimuli increase. Small neurons also have a significantly larger increase in their OFF currents than Large neurons with increasing ramp stimuli, as demonstrated by their greater *m* value (Supplementary Figure 2B). However, because the *b* value for Small neurons (1.905) is smaller than that of the Large neurons (2.194), the OFF current for Large neurons will consistently stay higher than that of Small neurons across the ramp stimuli that we tested (0−12 pA/ms) and is consistent with the data presented here that Large neurons have a longer duration of spiking than Small neurons. This means that the constant 10*^*b*^* also dictates the spiking properties of not only Large and Small neurons, but also those of Top and Bottom neurons (Figures 6C, 7E). Some of the properties that can contribute to the *b* and *m* parameters of each neuron include that neuron’s action potential threshold (rheobase – Figure 3G), I_Na_ properties, including inactivation (Figures 4E,G), Na_v_ density (Zengel et al., 1985; Sengupta et al., 2013) and distribution throughout the neuron (Trimmer and Rhodes, 2004; Kress and Mennerick, 2009), K^+^ current properties, including the fast-activating and inactivating A-type current (Iseppe et al., 2016) and the non-inactivating M-current (Nai et al., 2011; Li et al., 2015), the I_H_ (Figure 5; Pignatelli et al., 2013), and further biophysical properties. Some of the differences between Top and Bottom neurons may also come from morphological properties, including possessing an axon/AIS (Chand et al., 2015; Galliano et al., 2018) and the growing classification of DA neurons in the OB (Kosaka et al., 2019), among other factors.

### Do Olfactory Bulb Dopamine Neurons Act as High-Pass Filters?

Which spiking pattern is more effective at releasing neurotransmitter depends on the presynaptic plasticity that occurs in the DA neuron’s presynaptic terminals. If the synapses facilitate, then high-frequency bursts of activity are likely more effective. However, if depletion of the readily releasable vesicle pool predominates, then the low-frequency spike trains could be more effective. The efficacy of the response of DA neurons to ramp input thus raises several questions. Are OB DA neurons dependent on action potentials for DA release, and what are the most effective stimuli for inducing transmitter release from these OB DA neurons? How can these gating mechanisms contribute to functionality of OB DA neurons?

First, because OB DA release can be evoked by a single action potential (Borisovska et al., 2013), OB DA neurons receive excitatory synaptic input (Hayar et al., 2004), and their synaptic activity increases after depolarization (Baker, 1986; Nickell et al., 1994; Hsia et al., 1999; Berkowicz and Trombley, 2000; Ennis et al., 2001; Davila et al., 2003; Liu et al., 2013; Vaaga et al., 2017), it is likely that exocytosis of synaptic vesicles is triggered by electrical impulses. These levels of release would likely differ depending on the time of the day, with higher levels in the daytime and lower levels in the nighttime of rodents (Corthell et al., 2013).

Unlike the midbrain DA neurons (Suaud-Chagny et al., 1992; Suaud-Chagny, 2004; Zhang and Sulzer, 2004; Ito and Schuman, 2007; Zhang et al., 2009; Covey et al., 2016), to the best of our knowledge, there is no direct evidence to suggest that OB DA neurons are more sensitive to stronger stimuli. Rather our data combined with the functionality of these neurons provide support for the notion that they are more sensitive to weaker stimuli. Because OB DA neurons are inhibitory, they may filter out the background, tonic odors. In the context of the OB, this suggests that DA neurons may act as high-pass filters to allow stronger odor signals to be processed by the main output neurons (Korshunov et al., 2017). A similar hypothesis was described for the function of calretinin PGCs, which are also single spikers (Iseppe et al., 2016).

Whether DA neurons may act as high-pass filters depends on whether these neurons stop releasing transmitter during depolarization block. When these neurons receive a large enough stimulus, they will revert to inactivity, which is characterized by depolarization block (plateau, non-spiking phase that can be distinguished in Figures 3B,C,F). Does this inactivity mean that OB DA neurons can no longer be synaptically active? During depolarization block, these neurons have a depolarized membrane potential of about −40 to –30 mV. This depolarization could activate Ca_v_ channels that are necessary for inducing a synaptic cascade, thus releasing DA and GABA. If this is the case, then these neurons can still be synaptically active, even though they are quiescent in terms of their somatic action potentials. This would especially be likely if a somatic action potential/depolarization block is proximal to the Ca_v_ of dendrites, causing a dendritic release of transmitters. However, depolarization block causing transmitter release may not be as likely for DA neurons expressing an axon. Without somatic action potentials, saltatory conduction in the nodes of Ranvier of the axon may not be possible. If saltatory conduction still occurs during depolarization block, then we would expect to record back-propagating action potentials during depolarization block. Additionally, a simulated study shows that high-frequency stimulations of axons will cause partial depolarization block (Guo et al., 2018). Therefore, since there is/are a subpopulation of OB DA neurons that do express an axon (Galliano et al., 2018; Kosaka et al., 2019), and because DAergic projections can span up to 1 mm (Kiyokage et al., 2010), it is unlikely that sustained depolarization block will cause synaptic release at the axonal terminals of a subset of OB DA neurons. Future voltammetry studies, which can measure DA release from dendrites and axon terminals, while simultaneously recording depolarization block in soma, may be an effective approach for answering this question.

In the OB, DA release causes a presynaptic inhibition of OSNs via the D_2_ receptor, effectively decreasing excitatory input onto and from the M/TCs (Nickell et al., 1994; Hsia et al., 1999; Berkowicz and Trombley, 2000; Ennis et al., 2001; Davila et al., 2003; Liu et al., 2013; Vaaga et al., 2017). Perhaps, higher odor concentrations could inhibit DA neurons, as did the stronger depolarization stimuli (Figures 3E,F) and steeper ramps (Figures 7D–F). If these stronger odors bypass the DAergic network in the glomerulus, then these neurons may act as high-pass filters (Korshunov et al., 2017): actively inhibiting transmission of weak/ambient odors while being quiescent in the presence of stronger odors. Thus, the activity of OB DA neurons may increase odor discrimination through the D_2_ receptor (Tillerson et al., 2006) by inhibiting glutamate release from its intraglomerular OSNs and M/TCs, while having more complicated, temporal effects on its interglomerular targets (Liu et al., 2013).

### Clinical Implications

The increasing availability of transgenic mice over the past few decades has caused mice to assume a greater role in biomedical science compared to rats. However, the advent of transgenic rats such as this hTH-GFP rat line (Iacovitti et al., 2014) allows for further characterization of OB DA neurons from a different rodent species. This adds to the collective knowledge of the function of OB DA neurons, as well as how these neurons may be affected by neurodegenerative diseases such as PD, and is of particular interest to those in the fields of pathology and neurology. When afflicted with PD, the OB DA neurons of rats and people paradoxically increase in number (Huisman et al., 2004; Lelan et al., 2011; Mundiñano et al., 2011). A loss of olfaction – hyposmia and anosmia – precedes overt PD and can be a sign of the early stages of this disease (Doty et al., 1988; Berendse et al., 2001; Huisman et al., 2004; Ponsen et al., 2004; Ross et al., 2008). This hyposmia and anosmia is possibly due to increased inhibition from the greater number of DA-GABA neurons present in the affected OBs of PD patients (Alizadeh et al., 2015). In some rodent models of PD, rats (but not mice) appear to display Parkinsonian motor deficits more akin to the symptomology in humans (Ellenbroek and Youn, 2016). Our finding that OB DA neurons do not spontaneously spike in rats (Figure 3A), while they do in mice (Pignatelli et al., 2005; Puopolo et al., 2005), suggest biophysical differences that may be important in the function of the neurons in odor discrimination. Therefore, clarifying the function of DA neurons in mammalian, including human, OBs and investigating potential species differences may facilitate the successful design of clinical trials and treatments for olfactory dysfunction as well as the early detection of neurodegenerative disorders.

## Data Availability Statement

The datasets generated for this study are available on request to the corresponding author.

## Ethics Statement

The animal study was reviewed and approved by the Florida State University Animal Care and Use Committee.

## Author Contributions

KK, LB, RB, and PT designed the experiments and analyzed the data. KK performed the experiments, collected the data, and wrote the first draft of the manuscript. All authors contributed to subsequent drafts.

## Conflict of Interest

The authors declare that the research was conducted in the absence of any commercial or financial relationships that could be construed as a potential conflict of interest.

## Abbreviations

aCSF: artificial cerebrospinal fluid
AIS: axon initial segment
Ca_v_: voltage-gated Ca^2+^ channels
Cd: cadmium
Cs: cesium
DA: dopamine
EPSP: excitatory postsynaptic potential
ETC: external tufted cell
EPL: external plexiform layer
GABA: γ-amino butyric acid
GAD: glutamic acid decarboxylase
GCL: granule cell layer
GL: glomerular layer
HCN: hyperpolarization-activated cyclic nucleotide-gated channel
hTH-GFP: tyrosine hydroxylase green fluorescent protein
I_Ca_^2+^: Ca^2+^ current
I_H_: H-current
I_*Na*_: Na^+^ current
IPI: interpulse interval
JGC: juxtaglomerular cell
M/TC: mitral/tufted cell
MCL: mitral cell layer
Na_v_: voltage-gated Na^+^ channels
OB: olfactory bulb
ONL: olfactory nerve layer
OSN: olfactory sensory neuron
P: postnatal day
PD: Parkinson’s disease
PGC: periglomerular cell
SAC: short-axon cell
SEM: standard error of the mean
TH: tyrosine hydroxylase
V_f_: final voltage
V_i_: initial voltage.

## References

[B1] Alizadeh R. Hassanzadeh G. Soleimani M. Joghataei M. T. Siavashi V. Khorgami Z. (2015). Gender and age related changes in number of dopaminergic neurons in adult human olfactory bulb. *J. Chem. Neuroanat.* 69 1–6. 10.1016/j.jchemneu.2015.07.003 26212581

[B2] Aungst J. L. Heyward P. M. Puche A. C. Karnup S. V. Hayar A. Szabo G. (2003). Centre-surrounding inhibition among olfactory bulb glomeruli. *Nature* 426 623–629. 10.1038/nature02185 14668854

[B3] Baker H. (1986). Species differences in the distribution of substance P and tyrosine hydroxylase immunoreactivity in the olfactory bulb. *J. Comp. Neurol.* 252 206–226. 10.1002/cne.902520206 2431012

[B4] Baker H. Liu N. Chun H. S. Saino S. Berlin R. Volpe B. T. (2001). Phenotypic differentiation during migration of dopaminergic progenitor cells to the olfactory bulb. *J. Neurosci.* 21 8505–8513. 10.1523/JNEUROSCI.21-21-08505.2001 11606639 PMC6762814

[B5] Baker H. Towle A. C. Margolis F. L. (1988). Differential afferent regulation of dopaminergic and GABAergic neurons in the mouse main olfactory bulb. *Brain Res.* 450 69–80. 10.1016/0006-8993(88)91545-4 2900047

[B6] Bardoni R. Magherini P. C. Belluzzi O. (1996a). Excitatory synapses in the glomerular triad of frog olfactory bulb *in vitro*. *Neuroreport* 7 1851–1855. 10.1097/00001756-199607290-00033 8905679

[B7] Bardoni R. Puopolo M. Magherini P. C. Belluzzi O. (1996b). Potassium currents in periglomerular cells of frog olfactory bulb in vitro. *Neurosci. Lett.* 210 95–98. 10.1016/0304-3940(96)12677-x 8783281

[B8] Berendse H. W. Booij J. Francot C. M. J. E. Bergmans P. L. M. Hijman R. Stoof J. C. (2001). Subclinical dopaminergic dysfunction in asymptomatic Parkinson’s disease patients’ relatives with a decreased sense of smell. *Ann. Neurol.* 50 34–41. 10.1002/ana.1049 11456307

[B9] Berkowicz D. A. Trombley P. Q. (2000). Dopaminergic modulation at the olfactory nerve synapse. *Brain Res.* 855 90–99. 10.1016/s0006-8993(99)02342-2 10650134

[B10] Berkowicz D. A. Trombley P. Q. Shepherd G. M. (1994). Evidence for glutamate as the olfactory receptor cell neurotransmitter. *J. Neurophysiol.* 71 2557–2561. 10.1152/jn.1994.71.6.2557 7931535

[B11] Betarbet R. Zigova T. Bakay R. A. Luskin M. B. (1996). Dopaminergic and GABAergic interneurons of the olfactory bulb are derived from the neonatal subventricular zone. *Int. J. Dev. Neurosci.* 14 921–930. 10.1016/s0736-5748(96)00066-4 9010735

[B12] Biel M. Wahl-Schott C. Michalakis S. Zong X. (2009). Hyperpolarization-activated cation channels: from genes to function. *Physiol. Rev.* 89 847–885. 10.1152/physrev.00029.2008 19584315

[B13] Blakemore L. J. Resasco M. Mercado M. A. Trombley P. Q. (2006). Evidence for Ca^2+^- permeable AMPA receptors in the olfactory bulb. *Am. J. Physiol. Cell Physiol.* 290 C925–C935. 10.1152/ajpcell.00392.2005 16267106

[B14] Borisovska M. Bensen A. L. Chong G. Westbrook G. L. (2013). Distinct modes of dopamine and GABA release in dual transmitter neuron. *J. Neurosci.* 33 1790–1796. 10.1523/JNEUROSCI.4342-12.2013 23365218 PMC3579514

[B15] Bywalez W. G. Ona-Jodar T. Lukas M. Ninkovic J. Egger V. (2017). Dendritic arborization patterns of small juxtaglomerular cell subtypes within the rodent olfactory bulb. *Front. Neuroanat.* 10:127. 10.3389/fnana.2016.00127 28163674 PMC5247448

[B16] Cave J. W. Baker H. (2009). Dopamine systems in the forebrain. *Adv. Exp. Med. Biol.* 651 15–35. 10.1007/978-1-4419-0322-8_2 19731547 PMC2779115

[B17] Chand A. N. Galliano E. Chesters R. A. Grubb M. S. (2015). A distinct subtype of dopaminergic interneuron displays inverted structural plasticity at the axon initial segment. *J. Neurosci.* 35 1573–1590. 10.1523/JNEUROSCI.3515-14.2015 25632134 PMC4308603

[B18] Cockerham R. Liu S. Cachope R. Kiyokage E. Cheer J. F. Shipley M. T. (2016). Subsecond regulation of synaptically released dopamine by COMT in the olfactory bulb. *J. Neurosci.* 36 7779–7785. 10.1523/JNEUROSCI.0658-16.2016 27445153 PMC4951580

[B19] Corthell J. T. Stathopoulos A. M. Watson C. C. Bertram R. Trombley P. Q. (2013). Olfactory bulb monoamine concentrations vary with time of day. *Neuroscience* 247 234–241. 10.1016/j.neuroscience.2013.05.040 23727009 PMC3722297

[B20] Covey D. P. Bunner K. D. Schuweiler D. R. Cheer J. F. Garris P. A. (2016). Amphetamine elevates nucleus accumbens dopamine via an action potential-dependent mechanism that is modulated by endocannabinoids. *Eur. J. Neurosci.* 43 1661–1673. 10.1111/ejn.13248 27038339 PMC5819353

[B21] Davila N. G. Blakemore L. B. Trombley P. Q. (2003). Dopamine modulates synaptic transmission between rat olfactory bulb neurons in culture. *J. Neurophysiol.* 90 395–404. 10.1152/jn.01058.2002 12611989

[B22] Doty R. L. Deems D. A. Stellar S. (1988). Olfactory dysfunction in Parkinsonism: a general deficit unrelated to neurologic signs, disease stage, or disease duration. *Neurology* 38 1237–1244. 10.1212/WNL.38.8.1237 3399075

[B23] Ellenbroek B. Youn J. (2016). Rodent models in neuroscience research: is it a rat race? *Dis. Model. Mech.* 9 1079–1087. 10.1242/dmm.026120 27736744 PMC5087838

[B24] Ennis M. Zhou F. M. Ciombor K. J. Aroniadou-Anderjaska V. Hayar A. Borrelli E. (2001). Dopamine D2 receptor-mediated presynaptic inhibition of olfactory nerve terminals. *J. Neurophysiol.* 86 2986–2997. 10.1152/jn.2001.86.6.2986 11731555

[B25] Ennis M. Zimmer L. A. Shipley M. T. (1996). Olfactory nerve stimulation activates rat mitral cells via NMDA and non-NMDA receptors *in vitro*. *Neuroreport* 7 989–992. 10.1097/00001756-199604100-00007 8804037

[B26] Gall C. M. Hendry S. H. Seroogy K. B. Jones E. G. Haycock J. W. (1987). Evidence for coexistence of GABA and dopamine in neurons of the rat olfactory bulb. *J. Comp. Neurol.* 266 307–318. 10.1002/cne.902660302 2891733

[B27] Galliano E. Franzoni E. Breton M. Chand A. N. Byrne D. J. Murthy V. N. (2018). Embryonic and postnatal neurogenesis produce functionally distinct subclasses of dopaminergic neuron. *eLife* 7:e32373. 10.7554/eLife.32373 29676260 PMC5935487

[B28] Gentet L. J. Stuart G. J. Clements J. D. (2000). Direct measurement of specific membrane capacitance in neurons. *Biophys. J.* 79 314–320. 10.1016/S0006-3495(00)76293-X 10866957 PMC1300935

[B29] Golgi C. (1875). Sulla fina struttura dei bulbi olfactorii (On the fine structure of the olfactory bulb). *Riv. Sper. Freniatr. Med. Leg.* 1 404–425.

[B30] Guo Z. Feng Z. Wang Y. Wei X. (2018). Simulation study of intermittent axonal block and desynchronization effect induced by high-frequency stimulation of electrical pulses. *Front. Neurosci.* 12:858. 10.3389/fnins.2018.00858 30524231 PMC6262085

[B31] Halász N. Johansson O. Hökfelt T. Ljunghahl A. Goldstein A. (1981). Immunohistochemical identification of two types of dopamine neuron in the rat olfactory bulb as seen by serial sectioning. *J. Neurocytol.* 10 251–259. 10.1007/bf01257970 6118395

[B32] Hayar A. Karnup S. Ennis M. Shipley M. T. (2004). External tufted cells: a major excitatory element that coordinates glomerular activity. *J. Neurosci.* 24 6676–6685. 10.1523/JNEUROSCI.1367-04.2004 15282270 PMC6729710

[B33] Hodgkin A. L. Huxley A. F. (1952). A quantitative description of membrane currents and its application to conduction and excitation in nerve. *J. Physiol.* 117 500–544. 10.1113/jphysiol.1952.sp004764 12991237 PMC1392413

[B34] Holohean A. M. Rodriguez C. A. Hackman J. C. Davidoff R. A. (1996). Voltage-gated calcium currents in whole-cell patch-clamped bullfrog dorsal root ganglion cells: effects of cell size and intracellular solutions. *Brain Res.* 711 138–145. 10.1016/0006-8993(95)01414-4 8680856

[B35] Hsia A. Y. Vincent J. D. Lledo P. M. (1999). Dopamine depresses synaptic inputs into the olfactory bulb. *J. Neurophysiol.* 82 1082–1085. 10.1152/jn.1999.82.2.1082 10444702

[B36] Huisman E. Uylings H. B. M. Hoogland P. V. (2004). A 100% increase of dopaminergic cells in the olfactory bulb may explain hyposmia in Parkinson’s disease. *Mov. Dis.* 19 687–692. 10.1002/mds.10713 15197709

[B37] Iacovitti L. Wei X. Cai J. Kostuk E. W. Lin R. Gorodinsky A. (2014). The hTH-GFP reporter rat model for the study of Parkinson’s disease. *PLoS One* 9:e113151. 10.1371/journal.pone.0113151 25462571 PMC4251919

[B38] Iseppe A. F. Pignatelli A. Belluzzi O. (2016). Calretinin-periglomerular interneurons in mice olfactory bulb: cells of few words. *Front. Cell. Neurosci.* 10:213. 10.3389/fncel.2016.00231 27774053 PMC5054022

[B39] Ito H. T. Schuman E. M. (2007). Frequency-dependent gating of synaptic transmission and plasticity by dopamine. *Front. Neural Circuits* 1:1. 10.3389/neuro.04.001.2007 18946543 PMC2526279

[B40] Keller A. Yagodin S. Araniadou-Anderjaska V. Zimmer L. A. Ennis M. Sheppard N. F. Jr. (1998). Functional organization of rat olfactory bulb glomeruli revealed by optical imaging. *J. Neurosci.* 18 2602–2612. 10.1523/JNEUROSCI.18-07-02602.1998 9502819 PMC6793098

[B41] Kiyokage E. Pan Y. Z. Shao Z. Kobayashi K. Szabo G. Yanagawa Y. (2010). Molecular identity of periglomerular and short axon cells. *J. Neurosci.* 30 1185–1196. 10.1523/JNEUROSCI.3497-09.2010 20089927 PMC3718026

[B42] Korshunov K. S. Blakemore L. J. Trombley P. Q. (2017). Dopamine: a modulator of circadian rhythms in the central nervous system. *Front. Cell. Neurosci.* 11:91. 10.3389/fncel.2017.00091 28420965 PMC5376559

[B43] Kosaka K. Aika Y. Toida K. Heizmann C. W. Hunziker W. Jacobowitz D. M. (1995). Chemically defined neuron groups and their subpopulations in the glomerular layer of the rat main olfactory bulb. *Neurosci. Res.* 23 73–88. 10.1016/0168-0102(95)90017-9 7501303

[B44] Kosaka K. Kosaka T. (2005). Synaptic organization of the glomerulus in the main olfactory bulb: compartments of the glomerulus and heterogeneity of periglomerular cells. *Anat. Sci. Int.* 80 80–90. 10.1111/j.1447-073x.2005.00092.x 15960313

[B45] Kosaka K. Kosaka T. (2007). Chemical properties of type 1 and type 2 periglomerular cells in the mouse olfactory bulb are different from those in the rat olfactory bulb. *Brain Res.* 1167 42–55. 10.1016/j.brainres.2007.04.087 17662264

[B46] Kosaka K. Toida K. Aika Y. Kosaka T. (1998). How simple is the organization of the olfactory glomerulus?: the heterogeneity of so-called periglomerular cells. *Neurosci. Res.* 30 101–110. 10.1016/s0168-0102(98)00002-9 9579643

[B47] Kosaka K. Toida K. Margolis F. L. Kosaka T. (1997). Chemically defined neuron group groups and their subpopulations in the glomerular layer of the rat main olfactory bulb – II. Prominent differences in the intraglomerular dendritic arborization and their relationship to olfactory nerve terminals. *Neuroscience* 76 775–786. 10.1016/s0306-4522(96)00308-9 9135050

[B48] Kosaka T. Hataguchi Y. Hama K. Nagatsu I. Wu J. Y. (1985). Coexistence of immunoreactivities for glutamate decarboxylase and tyrosine hydroxylase in some neurons in the periglomerular region of the rat main olfactory bulb: possible coexistence of gamma-aminobutyric acid (GABA) and dopamine. *Brain Res.* 343 166–171. 10.1016/0006-8993(85)91172-2 2864104

[B49] Kosaka T. Kosaka K. (2008). Tyrosine hydroxylase-positive GABAergic juxtaglomerular neurons are the main source of the interglomerular connections in the mouse main olfactory bulb. *Neurosci. Res.* 60 349–354. 10.1016/j.neures.2007.11.012 18206259

[B50] Kosaka T. Kosaka K. (2009). Two types of tyrosine hydroxylase positive GABAergic juxtaglomerular neurons in the mouse main olfactory bulb are different in their time of origin. *Neurosci. Res.* 64 436–441. 10.1016/j.neures.2009.04.018 19445978

[B51] Kosaka T. Kosaka K. (2011). “Interneurons” in the olfactory bulb revisited. *Neurosci. Res.* 69 93–99. 10.1016/j.neures.2010.10.002 20955739

[B52] Kosaka T. Kosaka K. (2016). Neuronal organization of the main olfactory bulb revisited. *Anat. Sci. Int.* 91 115–127. 10.1007/s12565-015-0309-7 26514846

[B53] Kosaka T. Pignatelli A. Kosaka K. (2019). Heterogeneity of tyrosine hydroxylase expressing neurons in the main olfactory bulb. *Neurosci. Res.* 10.1016/j.neures.2019.10.004 [Epub ahead of print]. 31629793

[B54] Kress G. J. Mennerick S. (2009). Action potential initiation and propagation: upstream influences on neurotransmission. *Neuroscience* 158 211–222. 10.1016/j.neuroscience.2008.03.021 18472347 PMC2661755

[B55] Lelan F. Boyer C. Thinard R. Remy S. Usal C. Tesson L. (2011). Effects of human alpha-synuclein A53T-A30P mutations on SVZ and local olfactory bulb cell proliferation in a transgenic rat model of Parkinson’s disease. *Parkinsons Dis.* 2011:987084. 10.4061/2011/987084 21766003 PMC3135113

[B56] Li G. Linster C. Cleland T. A. (2015). Functional differentiation of cholinergic and noradrenergic modulation in a biophysical model of olfactory bulb granule cells. *J. Neurophysiol.* 114 3177–3200. 10.1152/jn.00324.2015 26334007 PMC4686300

[B57] Liu S. Plachez C. Shao Z. Puche A. Shipley M. T. (2013). Olfactory bulb short axon cell release of GABA and dopamine produces a temporally biphasic inhibition-excitation response in external tufted cells. *J. Neurosci.* 33 2916–2926. 10.1523/JNEUROSCI.3607-12.2013 23407950 PMC3727441

[B58] Liu S. Puche A. C. Shipley M. T. (2016). The interglomerular circuit potently inhibits olfactory bulb output neurons by both direct and indirect pathways. *J. Neurosci.* 36 9604–9617. 10.1523/JNEUROSCI.1763-16.2016 27629712 PMC5039244

[B59] Maher B. J. Westbrook G. L. (2008). Co-transmission of dopamine and GABA in periglomerular cells. *J. Neurophysiol.* 99 1559–1564. 10.1152/jn.00636.2007 18216231

[B60] McLean J. H. Shipley M. T. (1988). Postmitotic, postmigrational expression of tyrosine hydroxylase in olfactory bulb dopaminergic neurons. *J. Neurosci.* 8 3658–3669. 10.1523/jneurosci.08-10-03658.1988 2461434 PMC6569587

[B61] McQuiston A. R. Katz L. C. (2001). Electrophysiology of interneurons in the glomerular layer of the rat olfactory bulb. *J. Neurophysiol.* 86 1899–1907. 10.1152/jn.2001.86.4.1899 11600649

[B62] Meredith F. L. Benke T. A. Rennie K. J. (2012). Hyperpolarization-activated current (*I*_H_) in vestibular calyx terminals: characterization and role in shaping postsynaptic events. *J. Assoc. Res. Otolaryngol.* 13 745–758. 10.1007/s10162-012-0342-3 22825486 PMC3505587

[B63] Michel C. B. Azevedo C. C. Desmadryl G. Puel J. L. Bourien J. Graham B. P. (2015). Identification and modelling of fast and slow *I*_H_ current components in vestibular ganglion neurons. *Eur. J. Neurosci.* 42 2867–2877. 10.1111/ejn.13021 26174408 PMC4986932

[B64] Mundiñano I. C. Caballero M. C. Ordóñez C. Hernandez M. DiCaudo C. Marcilla I. (2011). Increased dopaminergic cells and protein aggregates in the olfactory bulb of patients with neurodegenerative disorders. *Acta Neuropathol.* 122 61–74. 10.1007/s00401-011-0830-2 21553300

[B65] Nagayama S. Homma R. Imamura F. (2014). Neuronal organization of olfactory bulb circuits. *Front. Neural Circuits* 8:98. 10.3389/fncir.2014.00098 25232305 PMC4153298

[B66] Nai Q. Dong H. W. Linster C. Ennis M. (2011). Activation of α1 and α2 noradrenergic receptors exert opposing effects on excitability of main olfactory bulb granule cells. *Neuroscience* 169 882–892. 10.1016/j.neuroscience.2010.05.010 20466037 PMC2904409

[B67] Nickell W. T. Behbehani M. M. Shipley M. T. (1994). Evidence for GABAB-mediated inhibition of transmission from the olfactory nerve to mitral cells in the rat olfactory bulb. *Brain Res. Bull.* 35 119–123. 10.1016/0361-9230(94)90091-4 7953767

[B68] Panzanelli P. Fritschy J. M. Yanagawa Y. Obata K. Sassoe-Pognetto M. (2007). GABAergic phenotype of periglomerular cells in the rodent olfactory bulb. *J. Comp. Neurol.* 502 990–1002. 10.1002/cne.21356 17444497

[B69] Parrish-Aungst S. Shipley M. T. Erdelyi F. Szabo G. Puche A. C. (2007). Quantitative analysis of neuronal diversity in the mouse olfactory bulb. *J. Comp. Neurol.* 501 825–836. 10.1002/cne.21205 17311323

[B70] Pignatelli A. Ackman J. B. Vigetti D. Beltrami A. P. Zucchini S. Belluzzi O. (2009). A potential reservoir of immature dopaminergic replacement neurons in the adult mammalian olfactory bulb. *Eur. J. Physiol.* 457 899–915. 10.1007/s00424-008-0535-0 19011893

[B71] Pignatelli A. Belluzzi O. (2017). Dopaminergic neurons in the main olfactory bulb: an overview from an electrophysiological perspective. *Front. Neuroanat.* 11:7 10.3389/fnana.2017.00007 PMC5306133 28261065

[B72] Pignatelli A. Borin M. Iseppe A. F. Gambardella C. Belluzzi O. (2013). The h-current in periglomerular dopaminergic neurons of the mouse olfactory bulb. *PLoS One* 8:e56571. 10.1371/journal.pone.0056571 23418585 PMC3572079

[B73] Pignatelli A. Kobayashi K. Okano H. Belluzzi O. (2005). Functional properties of dopaminergic neurones in the mouse olfactory bulb. *J. Physiol.* 564 501–514. 10.1113/jphysiol.2005.084632 15731185 PMC1464431

[B74] Pinching A. J. Powell T. P. (1971). The neuron types of the glomerular layer of the olfactory bulb. *J. Cell Sci.* 9 305–345. 4108056 10.1242/jcs.9.2.305

[B75] Ponsen M. M. Stoffers D. Booij J. van Eck-Smit B. L. Wolters E. C. h. Berendse H. W. (2004). Idiopathic hyposmia as a preclinical sign of Parkinson’s disease. *Ann. Neurol.* 56 173–181. 10.1002/ana.20160 15293269

[B76] Puopolo M. Bean B. P. Raviola E. (2005). Spontaneous activity of isolated dopaminergic periglomerular cells of the main olfactory bulb. *J. Neurophysiol.* 94 3618–3627. 10.1152/jn.00225.2005 16033943

[B77] Puopolo M. Belluzzi O. (1998). Functional heterogeneity of periglomerular cells in the rat olfactory bulb. *Eur. J. Neurosci.* 10 1073–1083. 10.1046/j.1460-9568.1998.00115.x 9753175

[B78] Ross G. W. Petrovich H. Abbott R. D. Tanner C. M. Popper J. Masaki K. (2008). Association of olfactory dysfunction with risk for future Parkinson’s disease. *Ann. Neurol.* 63 167–173. 10.1002/ana.21291 18067173

[B79] Ross M. T. Flores D. Bertram R. Johnson F. Hyson R. L. (2017). Neuronal intrinsic physiology changes during development of a learned behavior. *eNeuro* 4:ENEURO.0297-17.2017. 10.1523/ENEURO.0297-17.2017 29062887 PMC5649544

[B80] Sengupta B. Faisal A. A. Laughlin S. B. Niven J. E. (2013). The effect of cell size and channel density on neuronal information encoding and energy efficiency. *J. Cereb. Blood Flow Metab.* 33 1465–1473. 10.1038/jcbfm.2013.103 23778164 PMC3764378

[B81] Shepherd G. M. (1972). Synaptic organization of the mammalian olfactory bulb. *Physiol. Rev.* 52 864–917. 10.1152/physrev.1972.52.4.864 4343762

[B82] Shepherd G. M. Greer C. A. Mazzarello P. Sassoe-Pognetto M. (2011). The first images of nerve cells: Golgi on the olfactory bulb 1875. *Brain Res. Rev.* 66 92–105. 10.1016/j.brainresrev.2010.09.009 20940020 PMC4465565

[B83] Suaud-Chagny M. F. (2004). In vivo monitoring of dopamine overflow in the central nervous system by amperometric techniques combined with carbon fibre electrodes. *Methods* 33 322–329. 10.1016/j.ymeth.2004.01.009 15183181

[B84] Suaud-Chagny M. F. Chergui K. Chouvet G. Gonon F. (1992). Relationship between dopamine release in the rat nucleus accumbens and the discharge activity of dopaminergic neurons during local *in vivo* application of amino acids in the ventral tegmental area. *Neuroscience* 49 63–72. 10.1016/0306-4522(92)90076-e 1357587

[B85] Tillerson J. L. Caudle W. M. Parent J. M. Gong C. Schallert T. Miller G. W. (2006). Olfactory discrimination deficits in mice lacking the dopamine transporter or the D2 dopamine receptor. *Behav. Brain Res.* 172 97–105. 10.1016/j.bbr.2006.04.025 16765459

[B86] Trimmer J. S. Rhodes K. J. (2004). Localization of voltage-gated ion channels in mammalian brain. *Annu. Rev. Physiol.* 66 477–519. 10.1146/annurev.physiol.66.032102.113328 14977411

[B87] Vaaga C. E. Yorhason J. T. Williams J. T. Westbrook G. L. (2017). Presynaptic gain control by endogenous cotransmission of dopamine and GABA in the olfactory bulb. *J. Neurophysiol.* 117 1163–1170. 10.1152/jn.00694.2016 28031402 PMC5340883

[B88] Wahl-Schott C. Biel M. (2009). HCN channels: structure, cellular regulation and physiological function. *Cell. Mol. Life Sci.* 66 470–494. 10.1007/s00018-008-8525-0 18953682 PMC11131499

[B89] Wei C. J. Linster C. Cleland T. A. (2006). Dopamine D_2_ receptor activation modulates perceived odor intensity. *Behav. Neurosci.* 120 393–400. 10.1037/0735-7044.120.2.393 16719703

[B90] Wilson D. A. Sullivan R. M. (1995). The D2 antagonist spiperone mimics the effects of olfactory deprivation on mitral/tufted cell odor response patterns. *J. Neurosci.* 15 5574–5581. 10.1523/jneurosci.15-08-05574.1995 7643202 PMC1885985

[B91] Zengel J. E. Reid S. A. Sypert G. W. Munson J. B. (1985). Membrane electrical properties and prediction of motor-unit type of medial gastrocnemius motoneurons in the cat. *J. Neurophysiol.* 53 1323–1344. 10.1152/jn.1985.53.5.1323 3839011

[B92] Zhang H. Sulzer D. (2004). Frequency-dependent modulation of dopamine release by nicotine. *Nat. Neurosci.* 7 581–582. 10.1038/nn1243 15146187

[B93] Zhang L. Doyon W. M. Clark J. J. Phillips P. E. Dani J. A. (2009). Controls of tonic and phasic dopamine transmission in the dorsal and ventral striatum. *Mol. Pharmacol.* 76 396–404. 10.1124/mol.109.056317 19460877 PMC2713129

