# Supplementary figures and images for: Spiking and Membrane Properties of Rat Olfactory Bulb Dopamine Neurons
Source: Front Cell Neurosci. 2020 Mar 20;14:60. doi: 10.3389/fncel.2020.00060 (PMC7100387; doi:10.3389/fncel.2020.00060)

**A**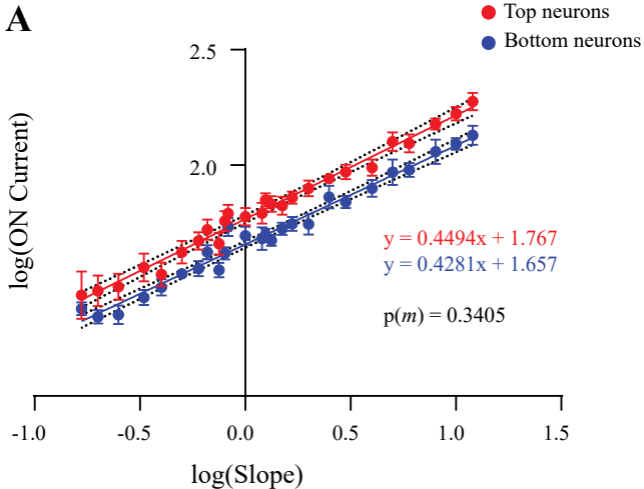**B**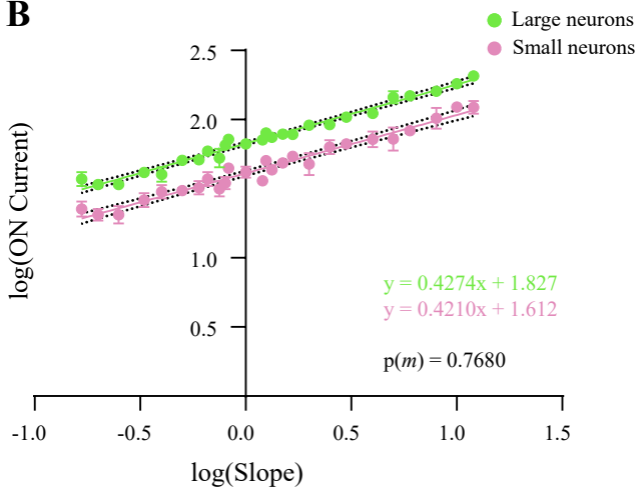

Supplement: Supplementary file 1 [file Data_Sheet_1.PDF]

**A**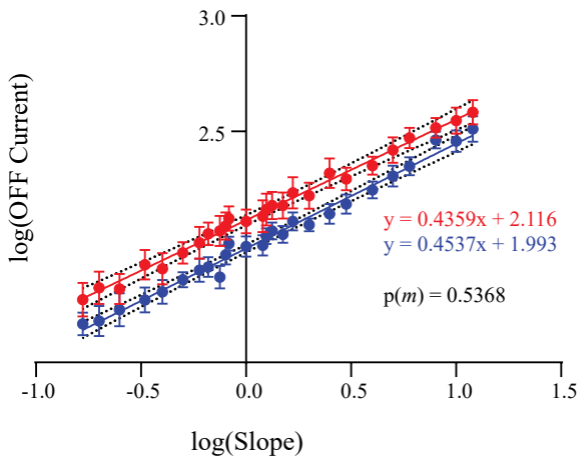**B**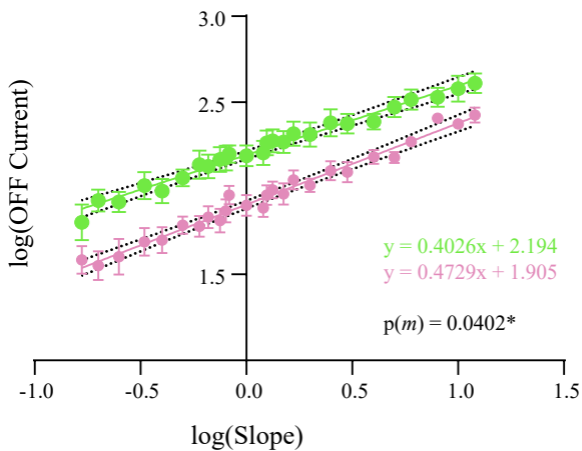

Supplement: Supplementary file 2 [file Data_Sheet_2.PDF]

**A**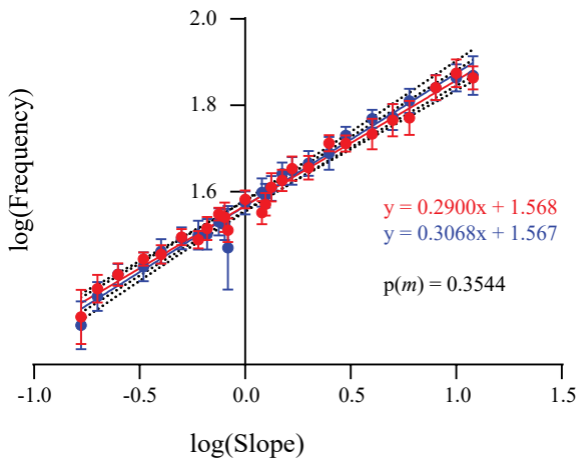**B**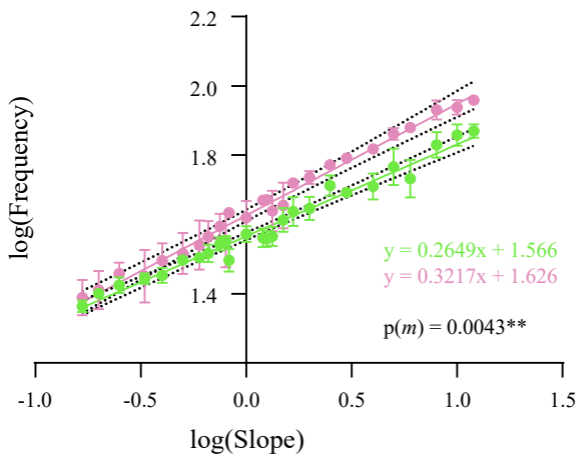

Supplement: Supplementary file 3 [file Data_Sheet_3.PDF]

**A**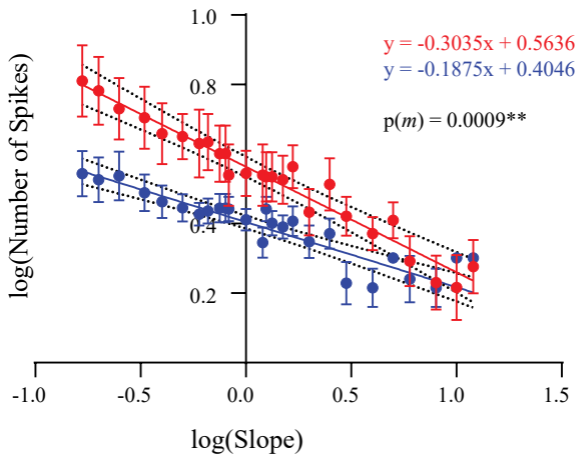**B**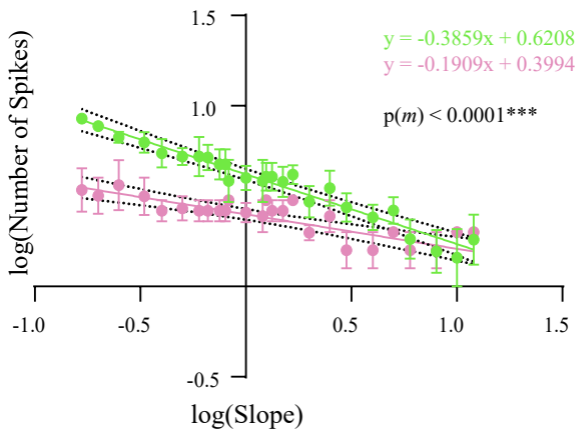

Supplement: Supplementary file 4 [file Data_Sheet_4.PDF]
