# Supplementary material for: Spiking and Membrane Properties of Rat Olfactory Bulb Dopamine Neurons
Source: Front Cell Neurosci. 2020 Mar 20;14:60. doi: 10.3389/fncel.2020.00060 (PMC7100387; doi:10.3389/fncel.2020.00060)
Supplement: Supplementary file 5 [file Data_Sheet_5.PDF]

Supplementary Figure Captions

Supplementary Figure 1

A common logarithm transformation of ramp slope and ON currents of Top and Bottom **(A)** and Large and Small **(B)** OB DA neurons. The linear equations used to compare for difference between the slopes of two lines and the resulting p-values are also displayed. From these equations, the derived power functions (defined as $y = 10^{b} \cdot x^{m}$, where *b* is the y-intercept of the log-log plot and *m* is the slope of the line in the log-log plot) are $y = 10^{1.767} \cdot x^{0.4494}$ for Top neurons, $y = 10^{1.657} \cdot x^{0.4281}$ for Bottom neurons, $y = 10^{1.827} \cdot x^{0.4274}$ for Large neurons, $y = 10^{1.612} \cdot x^{0.4210}$ for Small neurons.

Supplementary Figure 2

A common logarithm transformation of ramp slope and OFF currents of Top and Bottom **(A)** and Large and Small **(B)** OB DA neurons. The linear equations used to compare for difference between the slopes of two lines and the resulting p-values are also displayed. From these equations, the derived power functions are $y = 10^{2.116} \cdot x^{0.4359}$ for Top neurons, $y = 10^{1.993} \cdot x^{0.4537}$ for Bottom neurons, $y = 10^{2.194} \cdot x^{0.4026}$ for Large neurons, $y = 10^{1.905} \cdot x^{0.4729}$ for Small neurons.

Supplementary Figure 3

A common logarithm transformation of ramp slope and spike frequency of Top and Bottom **(A)** and Large and Small **(B)** OB DA neurons. The linear equations used to compare for difference between the slopes of two lines and the resulting p-values are also displayed. From these equations, the derived power functions are $y = 10^{1.568} \cdot x^{0.29}$ for Top neurons, $y = 10^{1.567} \cdot x^{0.3068}$ for Bottom neurons, $y = 10^{1.566} \cdot x^{0.2649}$ for Large neurons, $y = 10^{1.626} \cdot x^{0.3217}$ for Small neurons.

Supplementary Figure 4

A common logarithm transformation of ramp slope and number of spikes of Top and Bottom **(A)** and Large and Small **(B)** OB DA neurons. The linear equations used to compare for difference between the slopes of two lines and the resulting p-values are also displayed. From these equations, the derived power functions are $y = 10^{0.5636} \cdot x^{-0.3035}$ for Top neurons, $y = 10^{0.4046} \cdot x^{-0.1875}$ for Bottom neurons, $y = 10^{0.6208} \cdot x^{-0.3859}$ for Large neurons, $y = 10^{0.3994} \cdot x^{-0.1909}$ for Small neurons.
